# Present and Future Drinking Water Security and Its Impacts on Maternities: A Multi-Scale Assessment of Sudan

**DOI:** 10.3390/ijerph20032204

**Published:** 2023-01-26

**Authors:** Vincent Simonin, Saeid Ashraf Vaghefi, Zeinab M. Abdelgadir, Dalya Eltayeb, Mohammed Ahmed M. Sidahmed, Jean-Pierre Monet, Nicolas Ray

**Affiliations:** 1 Institute for Environmental Sciences, University of Geneva, Bd Carl-Vogt 66, 1205 Geneva, Switzerland; 2 Department of Geography, University of Zurich, 8006 Zurich, Switzerland; 3 United Nations Office for Project Services (UNOPS), Port Sudan, Sudan; 4 Federal Ministry of Health, Khartoum, Sudan; 5 The United Nations Population Fund (UNFPA), Khartoum, Sudan; 6 The United Nations Population Fund (UNFPA), Technical Division, 605 3rd Ave, New York, NY 10158, USA; 7 GeoHealth Group, Institute of Global Health, University of Geneva, 9 Chemin des Mines, 1202 Geneva, Switzerland

**Keywords:** water security, geographic accessibility, maternal health, climate change, Sahel

## Abstract

Adequate access to drinking water for hydration and hygiene depends on many factors. We developed the Drinking Water Security Index (DWSI) to assess relative multifactorial drinking water security at different spatial and temporal scales. DWSI is a function of four key indicators of drinking water security: water quality, water accessibility, water continuity, and water availability. We built DWSI with a total of 10 variables and applied the new index in Sudan to assess historical and future drinking water security at state, local, and maternity levels. Analyses at the state level found that the Red Sea and River Nile states are most vulnerable, with the lowest DWSI for both historical and future periods. The 1 km^2^ pixel level analysis shows large differences in water security within the major states. Analyses at the maternity level showed that nearly 18.97 million people are affected by the 10% of maternities with the lowest DWSI, a number projected to increase by 60% by 2030. Current and future DWSI of maternities providing Emergency Obstetric and Newborn Care was assessed to identify those where urgent action is needed to ensure quality health care in water-secure conditions. This work provides useful information for stakeholders in the health and drinking water sectors in Sudan, to improve public health, reduce preventable mortality, and make the population more resilient to projected environmental changes.

## 1. Introduction

A sufficient amount of clean water close to the household is essential for hydration, personal hygiene (handwashing, bathing, and clothes washing) and domestic hygiene (food preparation, utensils washing, and dwelling cleaning) to avoid water-related diseases, such as microbiological diseases (fecal-oral route mainly), chemical diseases and vector-borne diseases [1]. However, about 2 billion people worldwide do not have access to safe drinking water, and nearly half of the population still has to leave their homes to fetch water, especially in developing countries [2]. Globally, about 2.4 million deaths (mainly among children under five in developing countries) could be prevented annually if all people practiced proper hygiene and had access to safe, reliable drinking water and sanitation [3]. According to Victora et al. [4], households without easy access to piped water were found to be 4.8 times more likely to have a child die from diarrhea than households with piped water. During childbirth and after delivery, access to clean water, sanitation, and hygiene influences maternal and perinatal health outcomes [5]. Women with poor water supplies have been shown to be about 1.75 more likely to experience maternal mortality than women with adequate water supplies [6], and handwashing with clean water by birth attendants and mothers has been reported to increase neonatal survival by up to 44% [7]. Adequate quantities of good quality water are therefore essential not only in households but also in public or private health facilities such as hospitals or clinics. This is stated in the UN Sustainable Development Goal (SDG) #6 (clean water and sanitation) and in particular in targets 6.1 and 6.2, which aim to achieve universal and equitable access to safe and affordable drinking water and adequate and equitable sanitation and hygiene for all [2].

To inform progress toward these SDG targets in low- and middle-income countries (LMICs), identify gaps, and make appropriate decisions, comprehensive assessments at multiple spatial and temporal scales are essential. Assessments of household and health facility drinking water supplies depend on many factors, namely water source, geographic accessibility, continuity, water safety and quality, quantity, cost, and affordability, all of which can vary over time and space depending on climate, social, and economic issues [8]. While water source determines water quality and continuity (i.e., stability of water supply over time), water availability and the time in which water can be fetched determine the amount of water used by households or health facilities for hydration and hygiene. In addition, the continuity of water supply is also very important, as it has a direct impact on hydration and health, forcing households or health facilities to seek continuous sources of water that are potentially unsafe. These factors can be summarized in the concept of water security, defined by UN-Water [9] as the capacity of a population to safeguard sustainable access to adequate quantities of acceptable quality water for sustaining livelihoods, human well-being, and socioeconomic development, and to ensure protection from water-related pollution and water-related disasters.

Sudan is one of the driest countries in the world, but also one of the most vulnerable to climate variability and change [10]. With less than 100 m^3^ per capita per year of renewable internal freshwater resources [11], this country is considered to be in a situation of absolute water scarcity, according to Falkenmark Water Stress Indicator [12,13]. Of the Sudanese population, 51.8% have at least a basic drinking water service (i.e., an improved source accessible within a round trip of 30 min). However, there is wide variation in water supply among Sudanese states, with very low access rates in Gadarif (27.6% of households), White Nile (32.8%), and Red Sea (33.2%) [14]. In addition, the average annual temperature in Sudan may increase by 2.7 °C by 2050 due to climate change [15], which will lead to an increase in intra-annual variability and the frequency of extreme climatic events such as droughts and floods [16].

The maternal mortality rate in Sudan was estimated at 295 (UI at 80%: 207 to 408) maternal deaths per 100,000 live births in 2017 [17]. To reduce maternal and neonatal mortality in the coming years, the Sudanese Federal Ministry of Health (FMoH), with support from the United Nations Population Fund (UNFPA), initiated a process in November 2018 to design a national network of emergency obstetric and newborn care (EmONC) facilities—167 health facilities were selected to provide EmONC with an estimated 92% of the population having access to the closest EmONC facility within 2 h of travel time. This process followed a well-established methodology [18] based on several facility-related criteria, such as geographic accessibility, staffing, type of hospital, and average number of deliveries, but without considering the drinking water safety of health facilities as an explicit criterion. However, more than 50% of health facilities with access to water had a primary water source other than piped water, and many facilities suffer from water supply interruptions [19]. Knowing the current status of water security in health facilities and how it might evolve in the coming decades due to climate change could provide valuable insights into the current status of the health system and its sustainability, and could be one aspect to guide future planning.

To capture the different dimensions of water security and integrate different quantifiable indicators into a single value that can be used for decision-making, the use of an index is recommended [20,21]. There are several indices for the holistic assessment of water security [22,23,24,25,26]. These indices bring together agricultural, industrial, population, and environmental water needs and assess physical water availability as well as water quality, accessibility of water to the population, water management issues, and economic aspects. However, these existing indices remain relatively difficult to interpret and require expert participation because of the extensive input data [27]. In LMICs, information availability and data quality may be low, which may preclude the use of existing indices and require the use of a simple index consisting of fewer indicators. Existing indices are also usually calculated at the country level and are not readily implementable at the subnational or local level. In addition, the concept of sustainability of water security is usually ignored. These shortcomings of existing indices are particularly detrimental to large LMICs such as Sudan with spatial heterogeneities in climate, population density, and water use [23].

The objective of this study is to address a methodological gap in how to assess drinking water security in LMIC countries by using openly available data to facilitate replication in other countries. By developing a new multi-factorial index of drinking water security at multiple levels, we aim to assess drinking water security in Sudan at the state, local, and health facility levels for two time periods that are representative of the current situation and the future situation considering projected climate change. We identify the states, regions, and health facilities with the greatest water insecurity and, among them, those that are part of the network of EmONC health facilities. By combining our results with health accessibility analysis, we also attempt to quantify the population exposed to vulnerable health facilities now and in the future.

## 2. Methods

### 2.1. Study Area—Sudan

**Figure 1:**
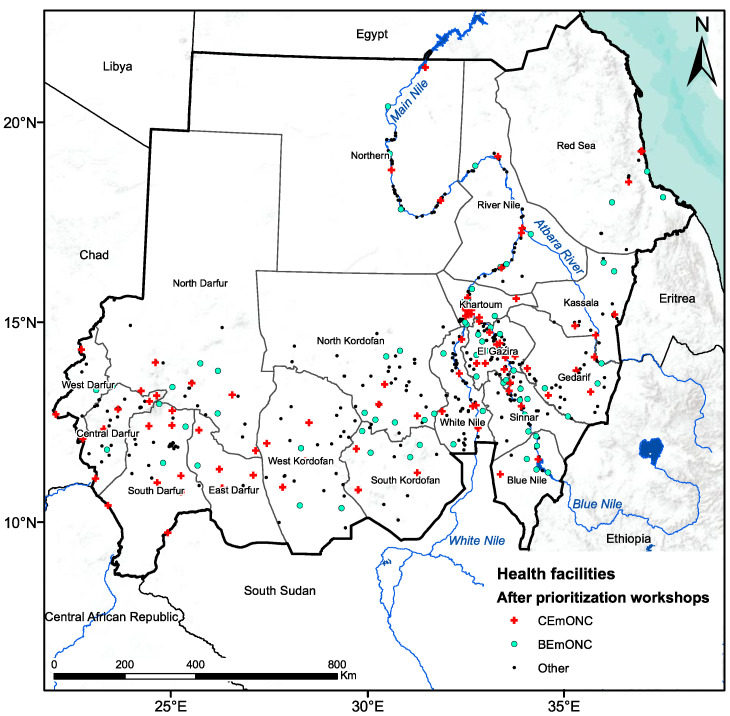
Map of Sudan, the 18 states, and the 631 health facilities that perform childbirth. CEmONC: Comprehensive emergency obstetric and newborn care; BEmONC: Basic emergency obstetric and newborn care.

Sudan is a large country in Northeast Africa with an area of 1.89 million km^2^ and a population of more than 45 million in 2022. The country is composed of 18 states (Figure 1). Annual rainfall varies from 25 mm in the desert and semi-arid desert to 700 mm in the southern part of Sudan and is limited to 2–4 months (July to October). Much of this water resource returns to the atmosphere through evapotranspiration, estimated at about 1700 mm in the south and 3000 mm in the center and north of the country [28].

Fifty percent of the country lies on groundwater sources ranging from 40 to 400 m depth [29]. The main groundwater basins are Nubian Sandstone formations aquifers (28% of the surface of the country), the Umm Rawaba basin, the basement complex, and the alluvial wadi-fill aquifers [30]. However, these resources are mostly non-renewable, because they are fossil water and deep aquifers unable to be refilled, except for the Nubian Nile aquifers recharging from the Nile river [31]. The long-term average of renewable groundwater resources is estimated at 4 billion m^3^ per year [32]. 

### 2.2. Composite Drinking Water Security Index (DWSI)

To assess water security at different spatial and temporal scales, we developed a new index, the Drinking Water Security Index (DWSI), consisting of four indicators with several quantifiable variables derived from global and freely accessible data. The DWSI is based on the work of Sullivan et al. [33], Assefa et al. [26], Aayog [24], and Aggarewal et al. [23], but focuses exclusively on drinking water, with fewer input data, and with higher spatial resolution. This index was developed to be used at three spatial levels, namely the state level, the 1 × 1 km pixel resolution level, and the health facility level. We apply the DWSI over two time periods of approximately 30 years each, defined by the available climate data: (1) a historical period from 1970 to 2006 and (2) a future period from 2020 to 2050 using five different climate scenarios.

The DWSI is a function of four key indicators of drinking water security: (1) water quality (water source and treatment), (2) water accessibility (fetching time and groundwater depth), (3) water continuity (drought events, interannual variability, and seasonality), and (4) water availability and quantity (water balance for groundwater recharge, groundwater storage, and groundwater productivity).

Each of the four numeric indicators is translated into several sub-indicators, depending on the scale/level of analysis (Table 1). The DWSI is calculated in relative terms and is therefore intended to assess differences in water security within a country when the data used are consistent across the country. All variables are first standardized between 0 (worst case) and 1 (best case) as follows:

(1) $$V_{ij}=\frac{v_{ij}-v_{jmin}}{v_{jmax}-v_{jmin}}\;\left(positive\;indicators\right)$$

(2) $$V_{ij}=\frac{v_{jmax}-v_{ij}}{v_{jmax}-v_{jmin}}\;\left(negative\;indicators\right)$$

where $V_{ij}$ is the standardized value of variable j in indicator i, $v_{ij}$ is the initial value of variable j in indicator i before standardization. $v_{jmax}$ and $v_{jmin}$ are, respectively, the maximum and minimum initial values found for the variable j.

The DWSI is calculated as a linear additive function to avoid the propagation of important errors and to determine the relative importance of each indicator [33]. We weighted the indicators equally and weighted each variable within an indicator equally because their relative importance to water security is unknown and cannot be clearly justified. Applying weights in such a case could lead to unnecessary complications and misinterpretation [26]. The general equation for the DWSI, which ensures that its values range from 0 (lowest value for water security score) to 100 (highest value), is as follows:

(3) $$DWSI=\overset{4}{\underset{i=1}{\sum }}\left[\left(\overset{n}{\underset{j=1}{\sum }}(V_{ij}\;\times \;w_{ij})\right)\times \;25\right]$$

where $V_{ji}$ is the standardized value assigned to variable j of indicator i, $w_{ij}$ is the weight assigned to variable j in indicator i, and n is the number of variables in indicator i. In our case of equal weighting, we apply $w_{ij}=1/n$ for each indicator. The multiplication by 25 ensures that *DWSI* ranges from 0 to 100 with equal weight for each of its four constituting normalized indicators.

### 2.3. Sources and Preparation of DWSI Variables

Quantitative and qualitative datasets (vector or raster) (from national surveys) were obtained from open-access sources, except for health facility locations, which were available upon request from the Ministry of Health. At the state level, variables associated with groundwater (depth, storage, productivity) and interpolated climate conditions (droughts, interannual precipitation variability, precipitation seasonality, and water balance ratio) were averaged within state boundaries prior to DWSI calculation. At the pixel level, the values of the variables “drinking water source”, “water treatment”, and “water acquisition time”, reported as a percentage of the state’s population, were applied to each pixel within the state boundaries. At the health facility level, the pixel values of the variables associated with groundwater and climate and containing the health facility were extracted to calculate the DWSI. Information on the dataset used is provided in Table 2. Below, we summarize the main methodological steps and explain the additional methodological details in Supplementary Materials.

#### 2.3.1. Water Supply Statistics at the State Level

For our variables related to water supply in Sudan, we used reports from the Multiple Indicator Cluster Survey (MICS) programme, which is typically published every 5 years. The Central Bureau of Statistics (CBS) of Sudan, in collaboration with the Federal Ministry of Health, UNICEF, UNFPA, and WHO, conducted four MICS in 1995, 2000, 2010, and 2014. The 1995 and 2000 surveys were not useful because indicators of water fetching time and water treatment were not available. We, therefore, used the 2010 and 2014 surveys [14,34] for informing the water supply statistics of the historical (1970–2006) and the future (2020–2050), respectively. Although these two surveys do not fall within our two chosen time frames, they represent the period before and after South Sudan’s independence in 2011 and are appropriate for capturing the multiple socioeconomic impacts prior to and following this event [41].

From the MICS, we extracted three variables:

1. Drinking water source: percentage of the population with access to an improved water source (i.e., designed to be protected from outside contamination, especially fecal contamination). The types of improved water sources are piped water (into dwelling, compound, yard, or plot, to neighbor, public tap/standpipe), tube well/borehole, protected well, protected spring, and rainwater collection.
2. Water treatment: percentage of the population using an unimproved water source but using adequate water treatment.
3. Water fetching time: percentage of the population with water fetching time of less than 30 min. Water fetching time includes waiting in line and can be from either an improved or unimproved water source.

#### 2.3.2. Maternities and Primary Water Source

We used the 2017 EmONC Need Assessment [19], a national cross-sectional survey of public and private hospitals and all mid-level facilities (thereafter, maternities) that provided childbirth services at the time of the survey. Geographic coordinates and information were available for a total of 691 maternities (19 referral/specialized hospitals, 66 state/general hospitals, 398 local/rural hospitals, 52 private or NGO maternity/general clinics, 156 public or NGO/private health centers). Of these, 167 were selected by FMoH in 2019 to become part of the EmONC facility network [35] (see Figure 1). These EmONC maternities fall into two categories: 78 are basic EmONC (BEmONC) and 89 are comprehensive EmONC (CEmONC), depending on the signal functions (health services) offered [42] (see Figure S1). In addition to providing BEmONC, CEmONC facilities can perform c-sections and blood transfusions. The methodology used to select the network of EmONC facilities is explained in general terms by Brun et al. (2020), with specifics for Sudan described in the EmONC technical report [35].

Maternity units that provide EmONC are reference health facilities for obstetric and newborn care and are able to provide the necessary emergency services in case of obstetric complications. Ensuring water security for these maternities is therefore particularly important to ensure quality health care. In addition to the many indicators related to staffing and obstetric activity, three water-related indicators were available for each maternity in the 2017 EmONC assessment report: “access to water”, “primary water source”, and “water interruption”. We used the primary water source information to create our DWSI “drinking water source” statistic by converting the six water source categories to a corresponding relative value (as follows in parentheses): Piped water (5), Hand pump (4), Well (3), River (2), Other source (1), No water (0). For our DWSI variable “water fetching time” at the facility level, all maternities with a water source nearby (i.e., source categories 5, 4, 3, 2, 1) receive a value of 1, while those with no water receive a value of 0. It was not possible to use data on water interruptions because 10% of the facilities were missing data on it and 60% of the facilities responded that they have frequent but short interruptions in water supply.

#### 2.3.3. Groundwater Dataset

Groundwater is the most important source of drinking water and domestic use in Sudan, with 80% of the Sudanese population relying on it [43]. We used data from MacDonald et al. [36] to determine our three DWSI variables, “groundwater depth”, “groundwater storage”, and “groundwater productivity” (see Supplementary Materials Figure S1). These three variables are critical for evaluating groundwater use because even if groundwater is near the surface and easily accessible, the aquifer may be depleted quickly or take some time to recharge due to its low permeability, which may affect water use.

We did not include surface water in the availability indicator of the DWSI because this resource accounts for less than 10% of the water used for domestic purposes [14]. Furthermore, access to surface water in Sudan is highly influenced by climatic, economic, and political factors, and it is difficult to predict how access to this resource will evolve in the future. In particular, the fact that the Nile Basin and its various sub-basins (Blue Nile, White Nile, Atbara River, and Main Nile), which cover 72% of the country, are shared by ten other countries is likely to lead to potential conflicts over surface water resources. For example, the newly filled Grand Ethiopian Renaissance Dam on the Blue Nile [44] has been controversial and the consequences of its operation for downstream states are not well known [45].

#### 2.3.4. Climate Data

To account for climatic conditions in DWSI, we used temperature and precipitation, both of which affect water resources and their use. In Sudan, weather stations are not well distributed and their data are not easily accessible and reliable. Therefore, we used daily weather data on precipitation and temperature (minimum and maximum) compiled by Abbaspour et al. [41] from monthly data with a resolution of 0.5°.

Historical weather data on 0.5° grid points, on a monthly basis from 1970 to 2006, are from Harris et al. [46]. Future climate projections are from five global circulation models (GCMs) developed as part of the Inter-Sectoral Impact Model Intercomparison Project (ISI-MIP5) for the period 1950 to 2099 and at a spatial resolution of 0.5° [47]. To reduce bias, we used the following five future GCMs: GFDL-ESM2M (NOAA/Geophysical Fluid Dynamics Laboratory), HadGEM2- ES (Met Office Hadley Center), IPSL-CM5A-LR (Institut Pierre-Simon Laplace), MIROC (AORI, NIES, and JAMSTEC), and NoerESM1-M (Norwegian Climate Center). These GCMs were run with one emission scenario, namely the Intergovernmental Panel on Climate Change (IPCC) Representative Concentration Pathways (RCP) 8.5. This scenario corresponds to an atmospheric concentration of 1330 ppm CO2eq and thus a radiative forcing of + 8.5 W/m2 by 2100 compared to the pre-industrial period without any stabilization [48].

The five future climate scenarios were corrected by statistical downscaling with observed climate records using the Climate Change Toolkit (CCT) developed by Vaghefi et al. [49]. This correction is used to correct climate models for systematic discrepancies between historical simulated data and historical observed data [47]. The average maximum temperature in Sudan ranges from 20 to 38 °C, with higher values in central Sudan and a predicted increase of about 2 °C in the future (Figures S2 and S3), given the average of GCMs. During the historical period, average annual precipitation ranged from less than 10 mm in northern Sudan to more than 600 mm in southern states (Figures S4 and S5). Climate projections disagree on whether precipitation will decrease or increase, particularly in northern Sudan, but there is consensus on increases in inter- and intra-annual variability.

Four DWSI variables are derived from these precipitation and temperature estimates: “Droughts” using the maximum consecutive dry days (i.e., <1 mm/day) per year, “Inter-annual variability” using the coefficients of variation of inter-annual precipitation, “Seasonal variability” using the coefficients of variation of intra-annual precipitation, and the “Water balance ratio”. Each of these variables was calculated for both the historical and future periods. Details on the calculation of each of these variables, the associated results for each time period, and the methodology for spatial interpolation can be found in Supplementary Materials.

### 2.4. Geographic Accessibility and Population Coverage of EmONC Facilities

To quantify the population living in the catchments of EmONC facilities that have a low water security index, we modeled the travel time of the population to the network of EmONC facilities using the Accessibility module of the open-source AccessMod 5 software [47]. Briefly, AccessMod uses a least-cost path algorithm to model the travel time required to reach the nearest EmONC facility from any location. The algorithm is applied to a travel impedance surface raster obtained by combining spatial data on elevation, land cover, roads, river networks, and other types of obstacles [47]. A travel scenario is then applied to this raster.

We used the same input datasets and travel scenarios as those used in the identification of the EmONC facility network conducted by FMoH and UNFPA in Sudan in 2019 [35]. The source, type, resolution, and year of each input dataset are shown in Table S1. Modes of travel (primarily motorized, with walking in some areas) and associated on- and off-road speeds were determined by consensus among national and state experts. This process was conducted independently for each state in dedicated regional workshops in 2019 to account for the unique characteristics of each state. The scenarios for each state can be found in Annex 4 of the EmONC facility network report [35].

Population coverage by the EmONC networks considered was assessed using the 2-h travel time threshold, i.e., the population that can reach the facility within 2 h. This 2-h threshold is the estimated average interval between the onset of postpartum hemorrhage, in the absence of medical interventions [44]. We used AccessMod’s Zonal Statistics module to determine the population within the 2 h threshold and AccessMod’s Geographic Coverage module to model the extent of the 2 h catchment areas for each EmONC facility. For this study, we limited our analysis to two groups of EmONC facilities, those in the 10% and 20% of all EmONC facilities with the lowest DWSI. The analysis was conducted using both historical and future DWSI.

The gridded population count of Sudan is from WorldPop [48]. The source data are from 2010, and to model the future population in 2030, we used the state-specific average growth rates from the Central Bureau of Statistics of Sudan for the period between 2008 and 2018. The population distribution grid for 2030 was calculated for each pixel of the 2010 WorldPop dataset using:

(4) $$P_{1}=\;P_{0}\;\cdot \;{\left(1+R_{x}\right)}^{n}$$

where $P_{1}$ and $P_{0}$ are the pixel values of the WorldPop raster population from 2030 and 2010, respectively. $R_{x}$ is the population growth rate specific to state x, and n=20, i.e., the number of years between 2010 and 2030.

## 3. Results

### 3.1. Assessment of DWSI at State Level

**Figure 2:**
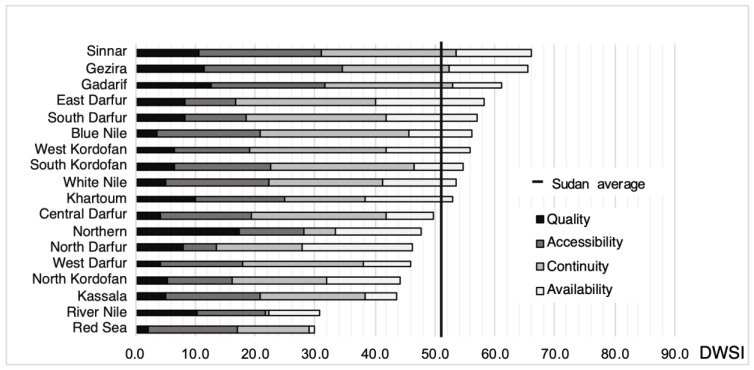
Ranking of the 18 states according to their historical DWSI. Vertical line indicates DWSI average across all states.

The calculated historical DWSI at the state level are shown in Figure 2 in rank order. The average DWSI is 51.1, ranging from 30.0 in the Red Sea to 66.2 in Sinnar. The eight states with the highest water insecurity, i.e., with a DWSI < 50, are located in the north and west of Sudan. One-third of Sudan’s population lived in these states in 2009, and they are the states with the highest proportion of the nomadic population (Table S2). Sinnar state has the highest historical DWSI, although only 60% of its population had access to improved water in 2010 (Table S2). This is because hydrogeologic conditions there are very favorable for pumping (large reservoir and high permeability) and rainfall during the historical period was less variable than in other states. Blue Nile has the lowest score on the water quality indicator, as less than 40% and 0.5% of its population had access to an improved water source and adequate water treatment in 2010, respectively (Table S2).

**Figure 3:**
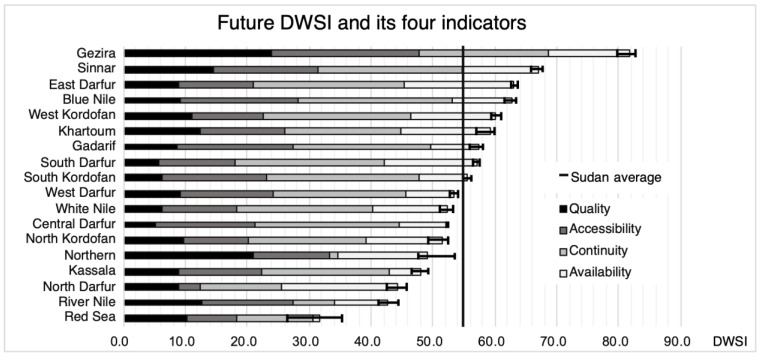
Ranking of the 18 states according to their future DWSI, with the indication of uncertainty ranges.

As shown in Figure 3, the future DWSI scores indicate increasing disparities in water security among states. DWSI scores range from 31.7 in the Red Sea to 81.7 in El Gazira (Figure 3). This may be the combined result of climate change, which is not uniform across Sudan, and differences in water resource management between states.

Regarding the two variables that compose the Quality indicator (“Drinking water source” and “Water treatment”), there have been improvements in most of the states between the two periods, but with a larger relative improvement for “Water treatment”. The average improvement of the standardized values for “Drinking water source” over all states is 13%, while there has been an average of 350% improvement of the standardized values for “Water treatment”.

The uncertainty interval is also shown in Figure 3. It is the range between the maximum and minimum values of the DWSI calculated using the five climate scenarios. With the exception of the northern states, such as the Northern and Red Sea, where the uncertainty about precipitation trends is larger in the five GCMs, the range is within 5% of the average. In Gezira state, the percentage of the population using an improved water source did not increase as much between 2010 and 2014 compared to the other states, but more than 11% of the population in this state who do not have an improved water source treated water before drinking (Table S2). Therefore, this state has the highest water quality and is by far the relatively most water-secure state for decades to come, with a very high DWSI score of about 80.

The Red Sea and River Nile states have the lowest DWSI for both the historical and future time periods, far below the other states. Both are located in the northern part of Sudan, where hydrogeological conditions are not optimal for the use of groundwater as a drinking water resource and where dry climatic conditions with low and highly variable rainfall prevail. Northern state is located at the same latitude as the Red Sea and the River Nile states and therefore has the same climatic and hydrogeological conditions and will also be most affected by climate change and its consequences for water resources. However, this state has a much higher historical and future DWSI value than the River Nile and the Red Sea because, since 2010, more than 90% of its population has access to a basic drinking water supply (improved water sources with a fetch time of less than 30 min). This allows this state to have higher scores for the water quality and accessibility variables, offsetting the negative impacts of current and future climate on drinking water security.

### 3.2. Assessment of DWSI at 1 km2 Pixel Level

**Figure 4:**
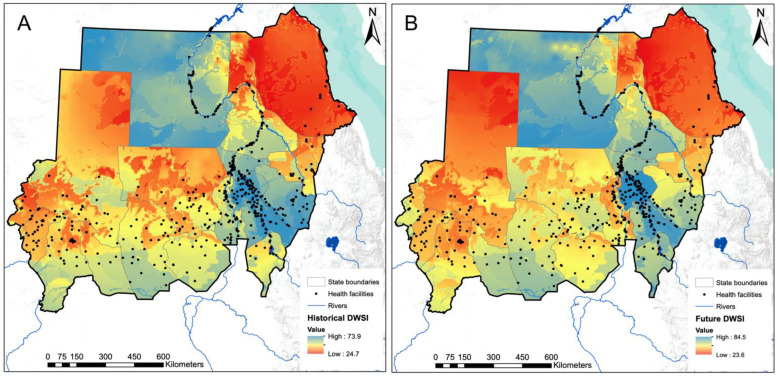
Assessment of the historical (**A**) and future (**B**) DWSI at a spatial resolution of 1 km^2^.

Figure 4 shows the variation in DWSI values at the 1 km pixel level, for both the historical and future time periods. The resulting maps are affected by state boundaries because the water source, water treatment, and water fetching time statistics are reported at the state level and therefore all pixels in a state receive the same value.

For both the historical and the future periods, northern and western states are more likely to suffer from water insecurity than southern and eastern states. A notable exception is the Northern state, where a large portion of the population has access to an improved water source that is less than 30 min fetching time, and therefore scores well on water quality and accessibility indicators.

These results also highlight the differences in drinking water security within each state. For example, the River Nile state has a wide range of DWSI scores, ranging from 33 to 65 and 36 to 71 for historical and future periods, respectively. Within this state, there is a latitudinal gradient in DWSI that results in very low DWSI values north of the Nile and a more water-secure region in the southern part. The states of Gezira and Central Darfur show the least variation in DWSI, likely due to their relatively small area and the associated lower variation in climate and groundwater conditions. Relatively, the results for the future period indicate that the northern parts of North Darfur, White Nile, Red Sea, and Gadarif are becoming more water insecure with very low DWSI.

The trend of precipitation and derived climate variables is more uncertain in the northern part of Sudan, which affects the reliability of the values of future DWSI in this region. Figure S10 shows this uncertainty at the pixel level by mapping the standard deviation of future DWSI over the values obtained by the five different GCMs. Greater uncertainty in future DWSI is observed in the Northern, Red Sea, and River Nile states.

### 3.3. Assessment of DWSI at Health Facility Level

**Figure 5:**
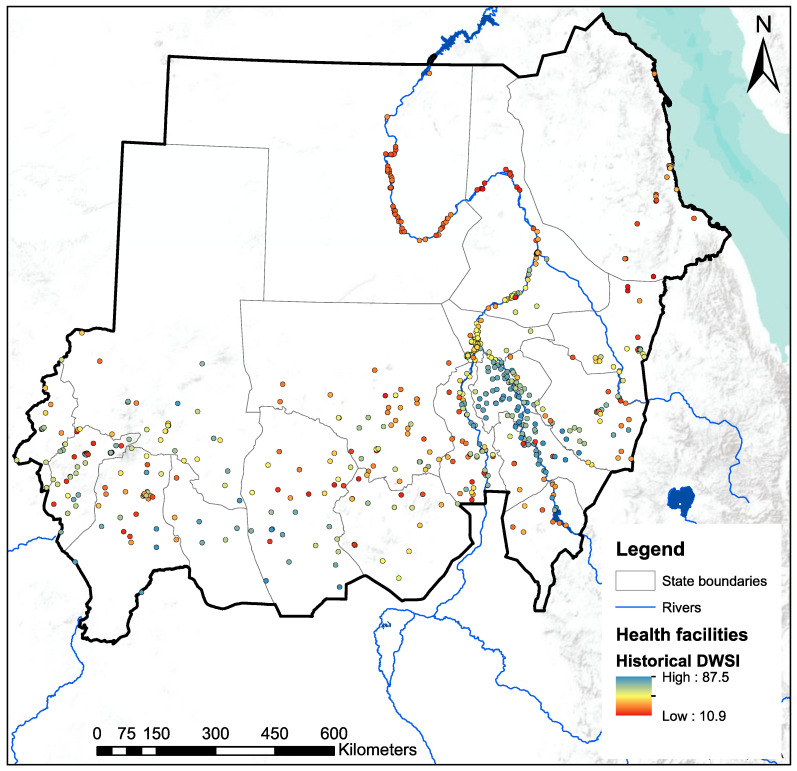
Historical DWSI at health facility level.

For the historical period, the DWSI of health facilities is shown in Figure 5. The average DWSI across all facilities is 68.1 and ranges from 10.9 to 87.5. All 37 health facilities that do not have a water source are in the bottom 10% of health facilities with the lowest DWSI. There is a concentration of health facilities with high water security in El Gazira and Sinnar states, which are also the two states with the highest historical DWSI (see Figure 4).

The DWSI values for the future period are shown in Figure S11 and range from 21.1 to 87.3. Figure S11 also shows the uncertainty of the DWSI values by indicating the relative mean deviation of the DWSI when calculated across the five different climate models.

### 3.4. Population Coverage of Facilities with Low DWSI

**Figure 6:**
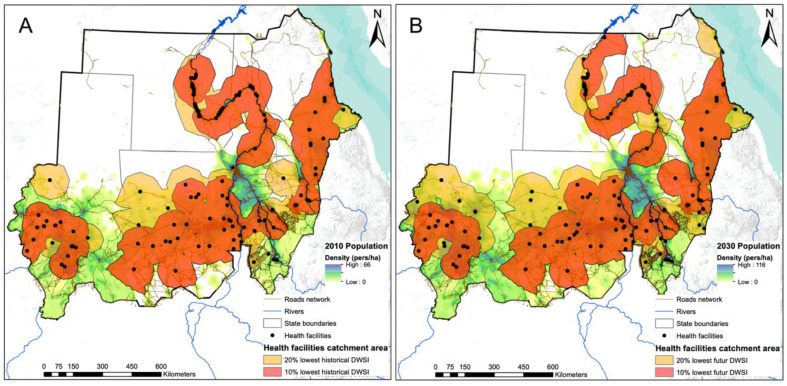
Historical (**A**) and future (**B**) catchment area of the 20% and 10% most water unsecure health facilities.

The 2 h maximum travel time catchments around the 20% of facilities (n = 140) with the lowest DWSI are shown in Figure 6 for both time periods. The population coverage of this group of facilities (i.e., the population living within the merged 2 h catchments) is 23.85 million people for the historical period. Considering only the 10% of facilities with the lowest DWSI (i.e., the red catchments in Figure 6), most of which are facilities without a water source, the population coverage for a maximum travel time of 2 h is about 18.97 million people.

In the future period, the 20% and 10% of health facilities with the lowest future DWSI will potentially cover a population of 39.90 and 30.41 million people, respectively (Figure 6, right panel). This increase in population coverage for the future period is due in part to natural demographic growth, but also to the fact that certain health facilities in densely populated regions will become relatively less water secure in the future, affecting a larger number of people. An example of this, shown in Figure 6, is two health facilities in the relatively densely populated south of North Darfur that were not among the 20% of health facilities with the lowest historical DWSI, but are included for the future period.

**Figure 7:**
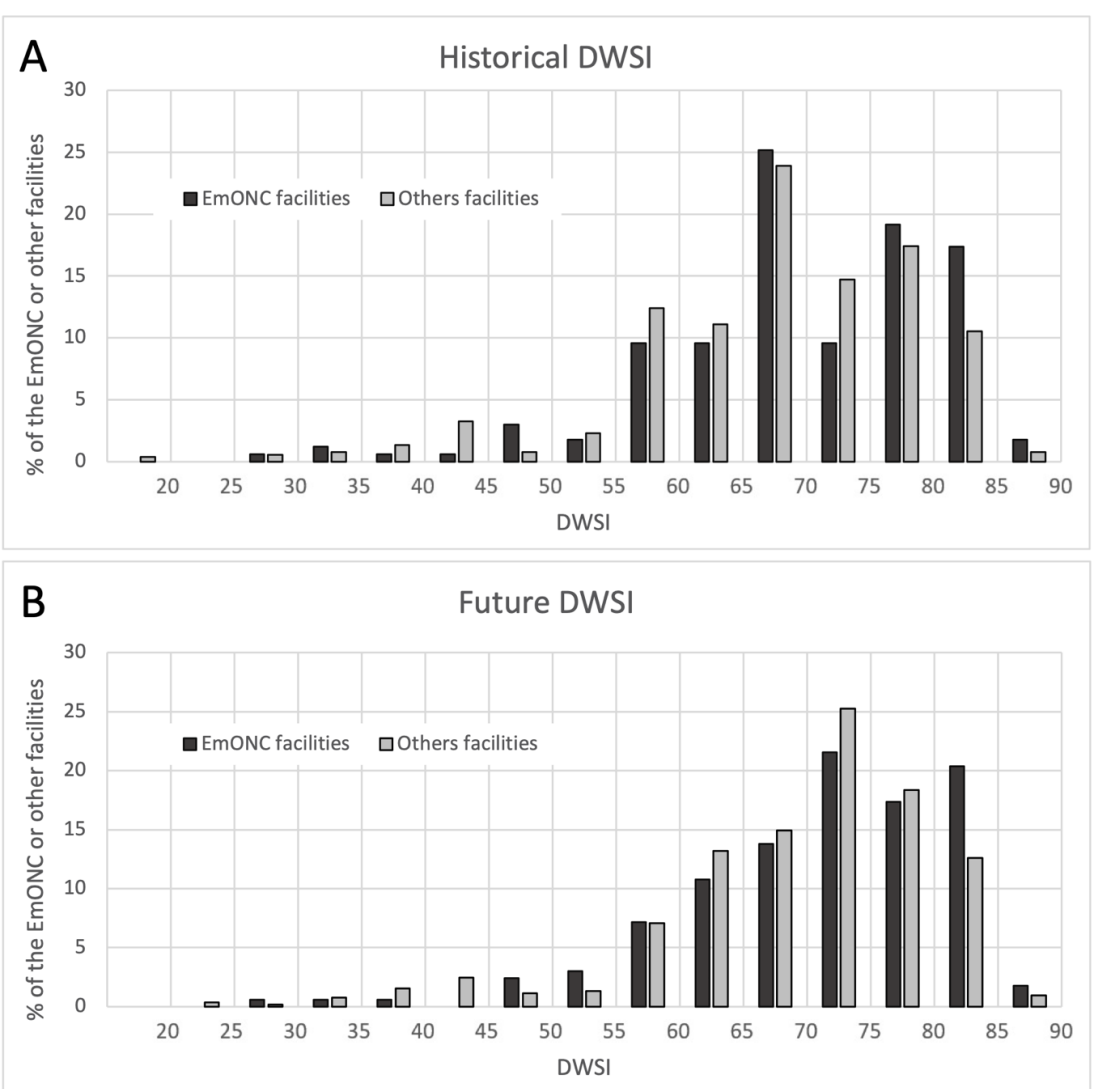
Distribution of DWSI values among EmONC facilities and other facilities, for the historical (**A**) and future (**B**) period.

Next, we focused only on the 167 facilities selected by FMoH and UNFPA to be part of the Emergency Obstetric and Newborn Care (EmONC) network. Figure 7 shows the distribution of health facilities by their DWSI and EmONC designation. Using a Wilcoxon test, we rejected the null hypothesis (at the 0.05 significance level) of the same DWSI distribution between the EmONC and “other facilities” groups for the historical period (*p* = 0.036). However, for the future period (Figure 7, right panel), there was no significant difference (*p* = 0.06) between the distributions of DWSI values for EmONC facilities and “other facilities”.

**Figure 8:**
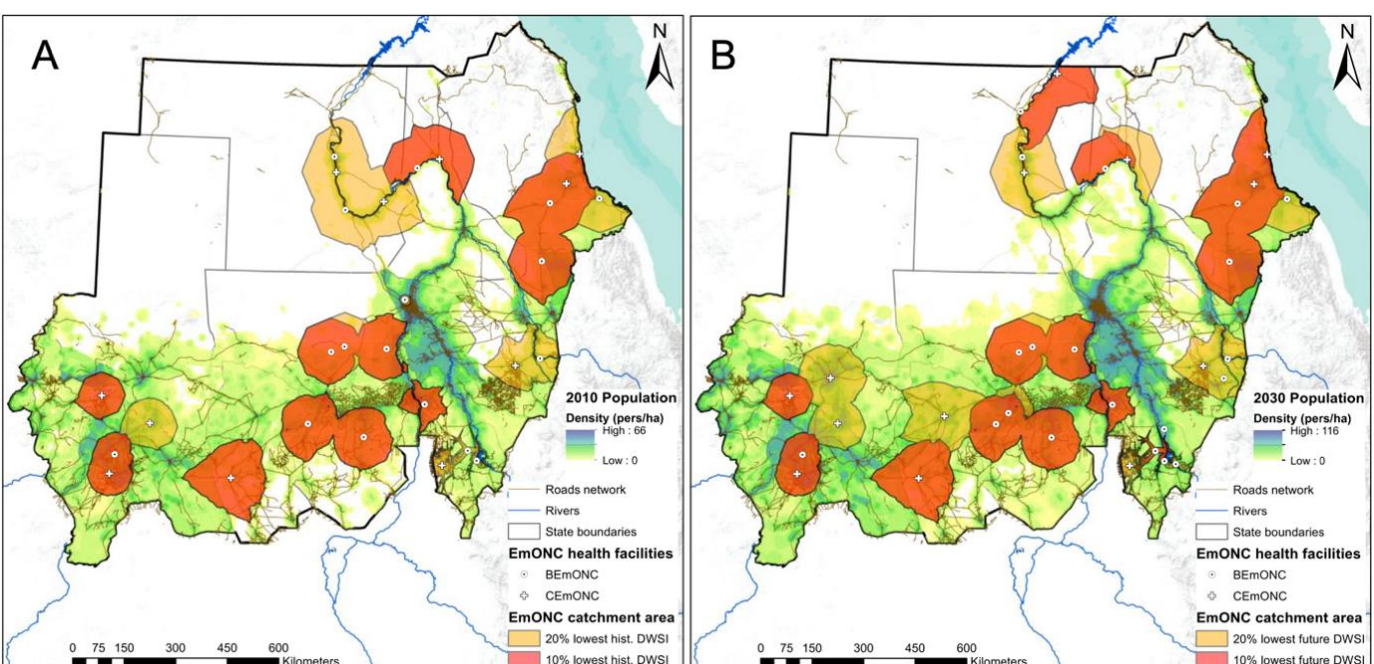
Historical (**A**) and future (**B**) catchment area of the EmONC facilities that are among the 20% and 10% of health facilities with the lowest DWSI.

We further divided EMONC facilities into two groups: basic EmONC facilities (BEmONC) (n = 78) and comprehensive EmONC facilities (CEmONC) (n = 89). The DWSI distribution between the two groups was found to be not significantly different using the Wilcoxon test, with *p* = 0.16 for both the historical and future periods. Of the 20% of health facilities with the lowest historical DWSI (n = 140), 18% (n = 25, with 15 BEmONC and 10 CeMONC) are part of the EmONC facility network. The population coverage of the 20% of EmONC facilities with the lowest DWSI is 12.88 million people when the merged 2-h catchment areas of these facilities are considered (Figure 8, left panel).

For the future period, of the 20% of health facilities with the lowest historical DWSI (n = 140), 24% (n = 33, with 20 BEmONC and 13 CeMONC) are part of the EmONC facility network. The population coverage of the 20% EmONC facilities with the lowest DWSi in the future period is 21.47 million people.

For the subset of the 10% of EmONC facilities with the lowest historical (n = 14) and future (n = 17) DWSI, the population coverage at 2 h is 8.66 million people for the historical period and 14.37 million people for the future period. As shown in Figure 8 (right panel), some of the EmONC facilities with the lowest future DWSI are located in more densely populated regions, particularly in southern North Darfur and northern West Kordofan.

## 4. Discussion

The first objective of this study was to present the methodology for creating a new relative index for assessing drinking water security at different spatial and temporal scales that addresses the needs identified in Sudan but can also be used in other countries. Our Drinking Water Security Index was found to be an appropriate tool for expressing some of the multiple dimensions of water security in a single value that can then be easily interpreted by policymakers and stakeholders in the water and health sectors. By focusing on drinking water, we limit the use of the index to human consumption and hygiene, leaving out water use for nature, industry, energy, agriculture, and other sectors. This limitation intentionally reduces the complexity of an index aimed at water security, especially in terms of the input data required. This limited dataset also has the advantage of being mostly freely accessible, which facilitates its use in various sectors.

In a large country like Sudan, with wide geographic variations in climatic conditions and composition of water and land resources, drinking water security also needs to be assessed at the local level to identify inequalities in the country and select regions or states where priority investments in the water sector need to be made. SDG 6.1 envisions achieving universal and equitable access to safe and affordable drinking water for all, i.e., not only at the domestic level but also for other institutions such as schools and health facilities. We have shown that DWSI can be appropriately calculated at the health facility level, and we discuss below the implications of the results for informing about the maternities that should be prioritized for improvement or close monitoring given the current or future impact of their local climate conditions.

### 4.1. Drinking Water Security at State and Pixel Levels

Our nationwide assessment of DWSI showed wide variation in drinking water security at the state level, as well as in scores on the four DWSI-forming indicators. For example, the Red Sea and River Nile states have the lowest DWSI for both the historical and future periods, but for different reasons. The Red Sea state has the lowest percentage of the population with access to an improved water source (27.4% in 2010 and 33.2% in 2014) and has unfavorable hydrogeological conditions, which is why it has low scores on both the quality and availability indicators. The River Nile River state is located in the region of Sudan with the highest rainfall variability (between years and seasonally) and suffers from long and recurrent droughts. Therefore, it has a very low score on the continuity indicator. Better water management should be promoted in these two states to avoid current and future water stress. However, the actions that should be taken in these two states differ according to our results. In the Red Sea, priority actions could focus on improving household water supplies and hydrogeological surveys to determine whether groundwater supplies are adequate. In River Nile, priority could be given to establishing safe and secure water storage facilities to mitigate the effects of rainfall variability. In addition, our pixel-level analysis showed that River Nile is the state with the widest range of DWSI, with values decreasing toward the north of the state. In this state, actions and investments to improve water storage could be focused on the northern population, where we found the lowest values of historical and future DWSI. Our findings are corroborated by the study of [10], who found that the most vulnerable states to water insecurity and climate change are the Red Sea, North Kordofan, and Kassala, three of the four states with the lowest historical DWSI.

Gadarif and North Darfur are the states with the largest decline in their DWSI and the largest decline in relative DWSI ranking compared to other states. Although these two states still have a higher future DWSI than the Red Sea and River Nile states, a more detailed analysis of their future drinking water security could be done to determine if preventive measures need to be taken to avoid a critical situation in the long term. In both states, the percentage of the population with a basic drinking water supply (improved water source with a fetching time of less than 30 min) decreased dramatically between 2010 (84% for Gadarif and 54% for North Darfur) and 2014 (72% for Gadarif and 21% for North Darfur). If this indicator does not improve in future Multiple Indicator Cluster Surveys, geographic accessibility to drinking water from safe sources should be a relevant priority measure to avoid lower drinking water insecurity in the coming decades.

### 4.2. Drinking Water Security at Facility Level

Our analysis at the health facility level showed that DWSI is decreasing toward the north and west of Sudan. Health facilities in El Gazira and Sinnar have historically been the most water secure, but in the future, their drinking water security will be relatively lower, with a greater decline in their DWSI compared to facilities in other states. Our estimates of population coverage at the 2 h travel time show that 20% of health facilities with the lowest DWSI serve 23.85 million people in Sudan who could potentially visit these water-insecure health facilities to receive health care. Our analyses show that this number will increase by 2.61% per year in the coming years, reaching 39.90 million people by 2030.

There was a significant difference in historical DWSI between facilities that are part of the EmONC network and those that are not. However, this difference will decrease in the future, making the prioritized EmONC network equally vulnerable to water insecurity. Of the 10% of health facilities with the lowest DWSI, 20% are part of the EmONC facility network. The latter proportion was unexpected as maternities in general, and especially those that can provide EmONC, are particularly dependent on the continuous provision of quality water to enable high levels of hygiene for mothers and their newborns. Our analyses have shown that in 2010, the set of 20 lowest DWSI EMONC facilities cover 8.66 million people. In 2030, 14.37 million people could be covered by EmONC facilities with very low water security, an annual growth of 2.56%.

The designation of the EmONC health facility network in Sudan took place in 2019 [35]. This process helped identify a number of maternities that can be prioritized to become fully functional EmONC facilities [18] and whose total number does not exceed the international norm of 5 EmONC facilities per 500,000 population. Our findings on the DWSI and its four facility-level factors were obtained after the designation of the EmONC facility network in Sudan and could not be considered during this process. However, EmONC prioritization processes should benefit from having information on facility-level drinking water security during prioritization workshops. Findings on those maternities whose drinking water supply is currently relatively unsafe, as well as information on DWSI in the future, could be added to current criteria (i.e., provision of EmONC signal functions, obstetric activity, 2-h population coverage, availability of health workers, quality of referrals, see [18]) to select the set of maternities to be part of the EmONC network. If maternity with a low current and/or future DWSI score is selected based on the current criteria, the four components of DWSI should be scrutinized to understand the factors that contribute most to the low DWSI score. The next step could be to either discuss and secure corrective actions to improve drinking water security at that facility or possibly prioritize an alternative maternity unit with a higher DWSI score.

More generally, decision-makers in health system strengthening and climate change adaptation can benefit from projected indicators of drinking water availability, continuity, and accessibility. Not only can they use this information at the level of existing facilities to improve drinking water security through various measures (e.g., improving water supply, water storage, or water treatment), but they can also benefit from continuous pixel-based DWSI mapping when new health services need to be located. In this case, sites with relatively higher current and future DWSI might be preferred if multiple alternatives are possible.

### 4.3. Limitations

Our study has some limitations related to the data and selection of indicators, as well as the methodology for DWSI calculation and catchment area modeling.

#### 4.3.1. Availability and Temporality of MICS Data

Because our goal in creating the DWSI was to use a methodology based on freely available and reliable data, some of the variables have weaknesses. The MICS statistics, which are compiled at the state level, significantly affect the pixel-level analysis and highlight the difference between rural and urban communities within the same state, but without available breakdown information by rural and urban communities within states. With the independence of South Sudan in 2011, it was not possible to find data on water availability and accessibility prior to 2011 recalculated with the current states and borders of Sudan. Therefore, for our historical DWSI with climate data from 1970 to 2006, we had to consider only the 2010 MICS statistics, whose representativeness for the entire period is necessarily limited. However, the 2010 MICS statistics predate South Sudan’s independence in 2011 and its multiple socioeconomic impacts.

In 2012, new states such as East Darfur, Central Darfur, and West Kordofan were created from the dissolution of another state. For our historical period, we decided to use the same data as for the state of origin, which may lead to a bias in the calculation of the index at the state and pixel level in these particular states.

The data for the variables that make up the index were not all representative of the same year, which may have affected the DWSI values. To improve the reliability of future DWSI estimates, the upcoming 2022 MICS report for Sudan [49] should be used for future studies aimed at obtaining the DWSI for the future period in Sudan.

#### 4.3.2. Health Facility Data

The health facility dataset is from the 2017 EmONC needs assessment and contains limited information on water supply in health facilities, as this was not the objective of this survey. For example, no data were available on potential water treatment at the facility. The water quality indicator was therefore calculated based solely on the water source variable. This means that a health facility that has a well or hand pump as its water source but adequately treats the water before consumption at the facility, will receive a lower water quality score than another health facility with piped water as its primary water source. Data were also not available on the facility’s storage capacity or alternative water sources that could be used during water shortages. This could reduce the impact of intra- and inter-annual fluctuations and provide the facility with better security than indicated in the DWSI. The water supply may sometimes depend on the continuity of the power supply at the facility level, but we did not consider this due to the lack of available data.

For the DWSI calculated for the future, we could not predict how the two water quality variables (drinking water source and water treatment) would evolve at the facility level. Some facilities may benefit from future FMoH investments to improve their water source and/or its treatment.

The three groundwater parameters are good indicators of water availability, but groundwater resources can be regularly affected by human activities, making them unsuitable for human consumption. In regions such as Sudan with an extended dry season, unconstrained groundwater demand could also exceed the availability of renewable groundwater, leading to aquifer drawdown and consequent increases in pumping costs, salinization, and possible soil subsidence, [36]. We could not consider possible contaminated or overexploited aquifers because of there being too little data on them for Sudan.

#### 4.3.3. DWSI Calculation

By design, our DWSI is a relative index, which has the advantage of not requiring thresholds to be set for its factors or the DWSI values above which water security is considered adequate. As a result, the DWSI cannot be used to measure how drinking water security is progressing for a particular state or health facility over the years because the respective DWSI values for each time period must be considered relative to the progress of the others. This could also limit the comparison of DWSI values between different countries for which different methods were used to determine the DWSI variables.

#### 4.3.4. Population Coverage

In capturing the population in health facilities with low DWSI scores, we did not consider other nearby health facilities with higher DWSI scores that might capture some of the population served in their catchment area. We also did not account for possible by-passing behavior [50,51] in which a portion of the population does not visit the nearest facility but travels a longer distance to reach another facility. Consequently, the calculated population coverages represent the maximum number of people who could be affected by these facilities and may have been overestimated in some cases.

## 5. Conclusions

This study was a first attempt to develop a Drinking Water Security Index based on openly available data and able to summarize the most important dimensions of drinking water security that can be assessed at different temporal and spatial scales. The results can warn of a risk situation and provide guidance to policymakers, but are in no way a substitute for additional field investigations. Further development of this index could eliminate some of the biases discussed, particularly the limitation of a relative index.

Through this study, we have identified the most vulnerable state or region in Sudan and identified appropriate interventions. At the facility level, we have shown that the network of EmONC facilities could face difficulties in the future in terms of access to potable water that could affect a large proportion of the country’s population. These findings may provide important information for policymakers and stakeholders in the health and water sectors in Sudan to improve public health and better prepare for future climatic changes. Future similar studies aimed at optimizing the EmONC network, and particularly in the drier countries of the Sahel, could benefit from additional insight coming from DWSI.

## Figures and Tables

**Table 1:** Indicators and sub-indicators (variables) of the DWSI with corresponding measurements, units, and characteristics.

Indicator	Variable	Measurements		Static (ST) Dynamic (DY) ^1^	Positive (+) Negative (−) ^2^
		At State and Pixel Level	At Health Facility (HF) Level		
Quality (Quali)	Drinking water source	% of population with improved water sources	Type of primary water source (5–1)	state: DY HF: ST	+
	Water treatment	% of population with unimproved water source but appropriate water treatment	*No available measurements*	state: DY HF: /	+
Accessibility (Acces)	Water fetching time	% of population at less than 30 min fetching time	Binary value, with (1) or without (0) water source	state: DY HF: ST	+
	Groundwater depth	Estimated depth to groundwater (m below ground level)		ST	−
Continuity (Conti)	Droughts	Maximum consecutive dry day (CDD) per year		DY	−
	Inter-annual variability	Coefficient of variation (CV) in annual precipitation (%)		DY	−
	Seasonal variability	Coefficient of variation (CV) in monthly precipitation (%)		DY	−
Availability (Avail)	Water balance ratio	Precipitation / Potential Evapotranspiration (-)		DY	+
	Groundwater storage	Water depth (m)		ST	+
	Groundwater productivity	Borehole yields expected (L/s)		ST	+

^1^ Dynamic variables are those that potentially change between the two periods, while the other variables are considered static. ^2^ Direction of variable influence on DWSI value: (−) are those for which lower values are better for drinking water security and (+) are those for which higher values are better.

**Table 2:** Database of the DWSI input data, initial format, and year of publication.

DATA TYPE	Source	Data Format	Representative Year	Publication Year
Water supply statistics at state level	Multiple Indicator Cluster Survey (MICS) [14,34]	Report and tables	2010/2014	2012/2016
Health facilities location and their primary water source	Sudan EmONC Need Assessment [19]	Vector and table	2017	2018
EmONC facility network	Identifying the national network of health facilities providing EmONC in the Republic of Sudan—Technical report [35]	Report and table	2019	2021
Groundwater resources (depth, storage, productivity)	Quantitative maps of groundwater resources in Africa [36]	Raster (5 × 5 km)	2011	2012
Administrative boundaries	OCHA Regional Office for Southern and Eastern Africa (HDX) [37]	Vector	2018	2018
Global Historical Climate Data	Climatic Research Unit (CRU) and dGen [38]	Tables (0.5° grid points)	1970–2006	2007
Global IPCC Climate Database	5 GCMs (GFDL, HadGEM, IPSL, MIROC, NoerESM) and 1 RCP (8.5) [39]	Tables (0.5° grid points)	2006–2099	2019
Observed water discharge	Global Runoff Data Centre (GRDC) and the Global River Discharge Database (RivDIS) [40]	Table	1900–1984	2022

## Data Availability

The Sudan EmONC Need Assessment is available from UNFPA-Sudan. All other input data sets are publicly available (sources are listed in Table 2). All output results in geospatial format are available from the corresponding author upon request.

## Supplementary Materials

The following supporting information can be downloaded at: https://www.mdpi.com/article/10.3390/ijerph20032204/s1, Figure S1: Quantitative maps of groundwater resources in Africa; Figure S2: Interpolation of historical and future maximal temperature. Figure S3: Historical maximal temperature and projected future maximal temperature according to each global circulation model. Figure S4: Interpolation of historical and future annual precipitation. Figure S5: Historical mean precipitation and projected future mean precipitation according to each global circulation model. Figure S6: Interpolation of historical and future average consecutive dry days per year. Figure S7: Interpolation of historical and future coefficient of variation (CV) of annual precipitation. Figure S8: Interpolation of historical and future coefficient of variation of monthly precipitation. Figure S9: Interpolation of historical and future water balance ratio calculated with annual precipitation and evapotranspiration. Figure S10: Standard deviation in the future DWSI over the values obtained by running the five different global circulation models. Figure S11: Future DWSI at health facility level with the uncertainty range of the future DWSI. Table S1: Source, type, format, resolution, and year of each input dataset; Table S2: Population and water supply statistics at state level. References [52,53,54,55,56,57,58,59,60,61,62,63,64,65,66,67,68,69,70,71,72,73] are cited in the supplementary materials.
