# Supplementary material for: Present and Future Drinking Water Security and Its Impacts on Maternities: A Multi-Scale Assessment of Sudan
Source: Int J Environ Res Public Health. 2023 Jan 26;20(3):2204. doi: 10.3390/ijerph20032204 (PMC9915021; doi:10.3390/ijerph20032204)
Supplement: Supplementary file 1 [file ijerph-20-02204-s001.zip › ijerph-2026321-supplementary.pdf]

Present and future drinking water security and its impacts on health facilities: a multi-scale assessment of Sudan

Vincent Simonin, Saeid Ashraf Vaghefi, Zeinab M. Abdelgadir, Dalya Eltayeb, Mohammed Ahmed M. Sidahmed, Jean-Pierre Monet, Nicolas Ray

Supplementary Materials

Detailed methodological steps

Water supply statistics at state level

From the 2010 and 2014 Multiple Indicator Cluster surveys [1,2], three variables were extracted at state level:

- Percentage of population with access to improved water source: this corresponds to the situation where water sources are designed to be protected from outside contamination, in particular by faecal matter. According to WHO [3], improved water source could be piped water (into dwelling, compound, yard or plot, to neighbor, public tap/standpipe), tube well/borehole, protected well, and protected spring. Unimproved water sources are: unprotected dug well, unprotected spring, cart with tank or drum, tanker truck, surface water, and bottled water.

- Percentage of population using unimproved water source but using appropriate water treatment: According to MICS [1,2] and WHO [3], the effective treatments of drinking water are boiling water, adding bleach or chlorine, water filter and solar disinfection.

- Percentage of population at less than 30 minutes water fetching time: the fetching time includes queuing, and this is from an improved or unimproved water source. Water quality decreases when water has to be transported and stored, as there is increasing opportunities and time for microbial contamination [4]. Within 1 km or 30 minutes of the total collection time, the quantity of water is at least 20 liters per person per day and can ensure the minimum consumption and hygiene needs [5].

In 2010, the states of East Darfur, Central Darfur and West Kordofan did not yet exist; they were created or reestablished after 2012 by the subdivision of South Darfur, West Darfur and South Kordofan respectively [6]. For these three new states, the same 2010 statistics as their state of origin have been used for our historical period.

Groundwater dataset

To inform our three DWSI variables "Groundwater depth", "Groundwater storage", and "Groundwater productivity", the following data from MacDonald et al. [7] were used, respectively:

- "Depth to aquifer" controls the accessibility and cost of assessing groundwater. When groundwater is deeper than 50m, it becomes difficult to extract it with a hand pump.
- "Groundwater storage" expressed as water depth in meters, is a combination of saturated thickness and effective porosity of aquifer and is a good indicator of water availability.
- "Aquifer productivity" indicates the boreholes yields that can be expected in different hydrogeological units and the amount of water that can be finally used.

Maps of these three variables and of Sudan geology are shown in Figure S1.

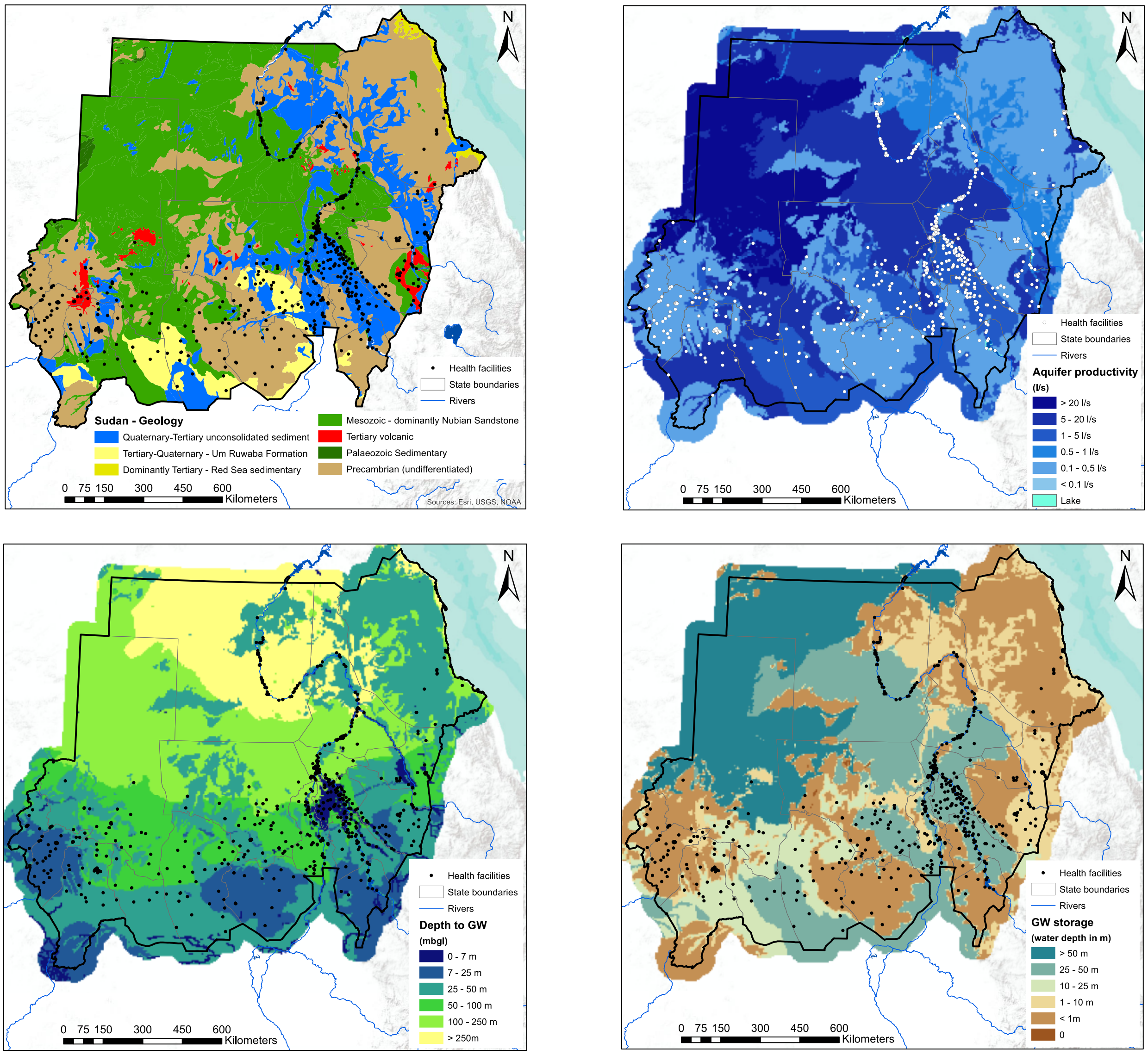

Figure S1 – Quantitative maps of groundwater resources in Africa from MacDonald et al. (2012), used for informing the groundwater-related DWSI variables

Climatic data

Figure S2 shows the interpolated values of average historical and future maximal temperatures over the five climatic models, and Figure S3 the future maximal temperatures for each climatic scenario. The Red Sea has a thermal influence on the continent since the state of Red Sea is subject to lower temperatures than the rest of the country located at the same latitudes. All models of climate change are predicting an increase in average temperatures, and all regions of Sudan will experience an increase of about 2°C in maximum temperature.
Figures S4 and S5 show the average annual precipitation ranging from less than 10 mm in northern Sudan to more than 600 mm in southern states.

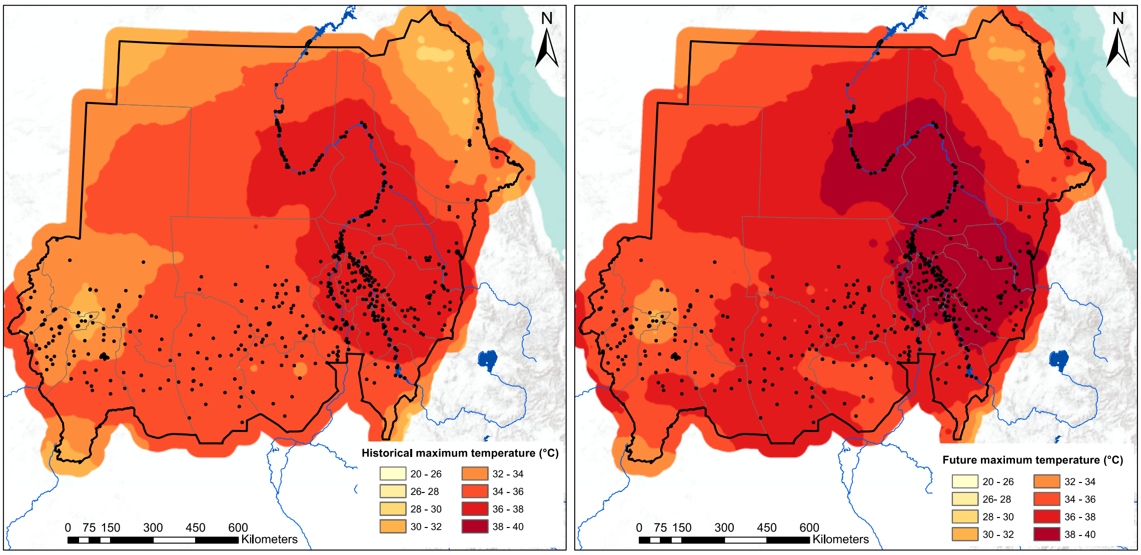

Figure S2 - Interpolation of historical (left) and future maximal temperature (right), at 0.5° resolution, with overlaid maternities (black dots)

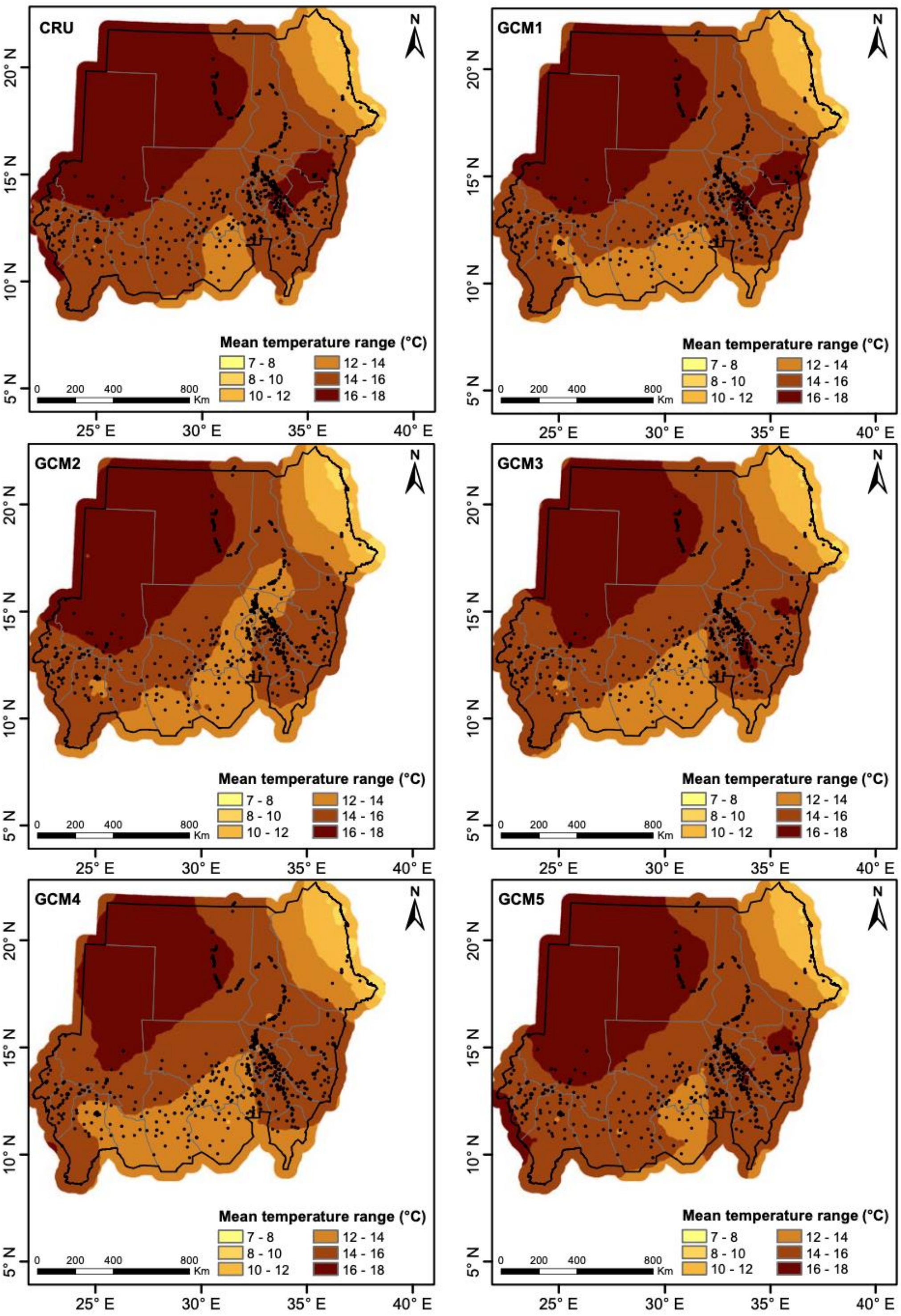

Figure S3 – Historical maximal temperature (CRU) and projected future maximal temperature according to each GCM, at 0.5° resolution, with overlaid maternities (black dots).

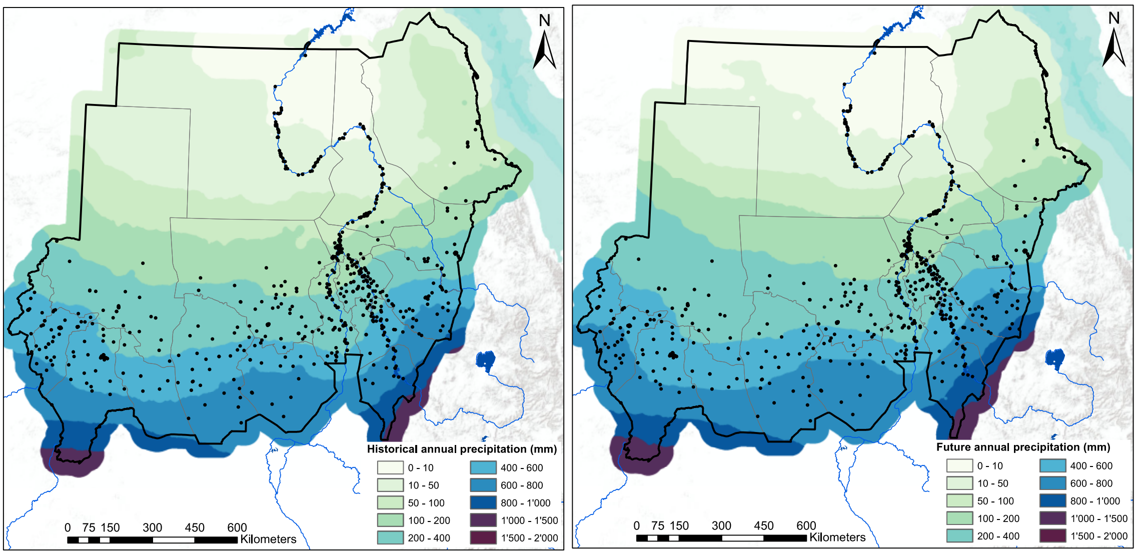

Figure S4 - Interpolation of historical (left) and future (right) annual precipitation (CRU and GCMs climate date at 0.5° grid points)

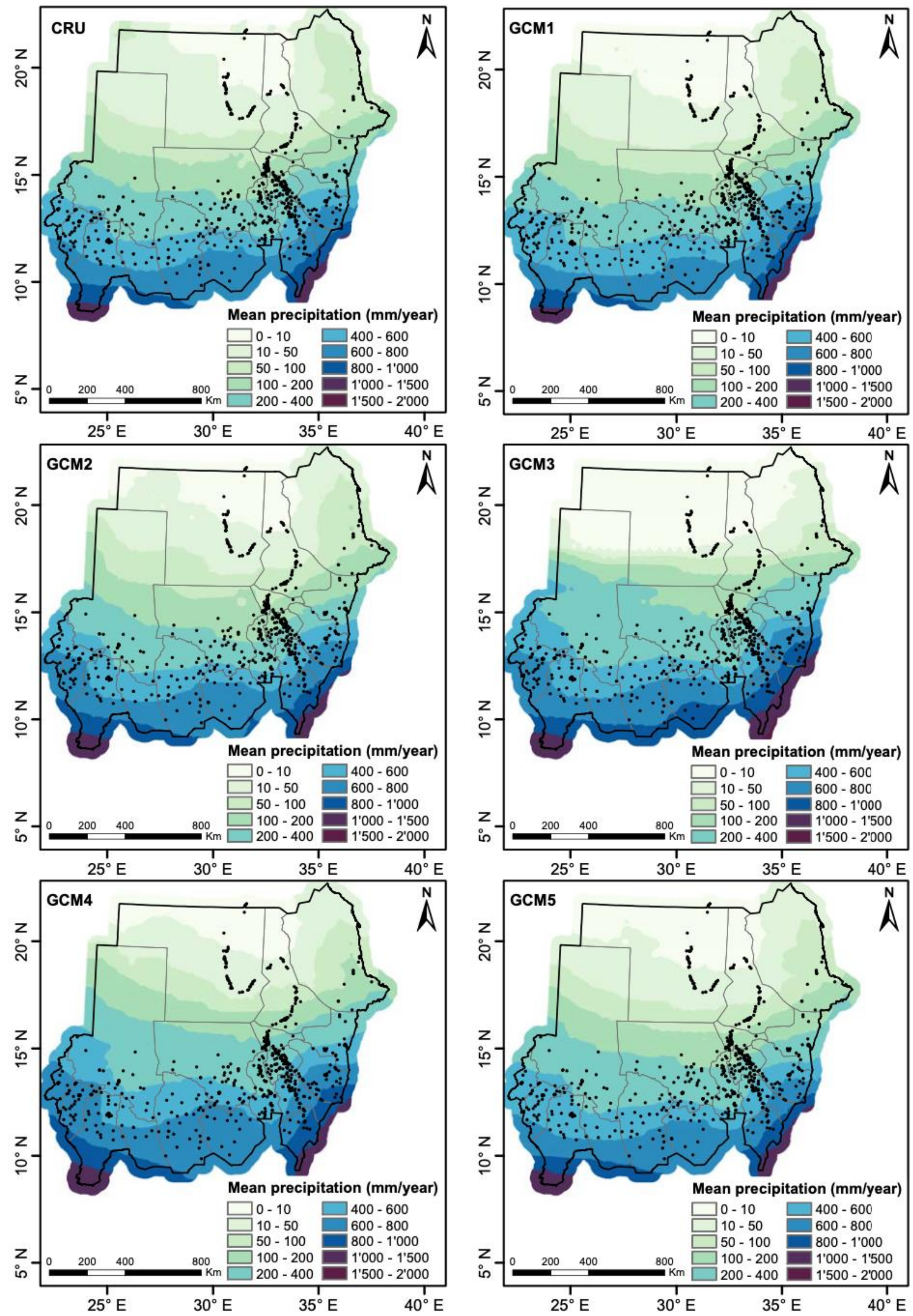

Figure S5 – Historical mean precipitation (CRU) and projected future mean precipitation according to each GCM, at 0.5° resolution, with overlaid maternities (black dots).

DWSI variable "Droughts"

This variable is based on the number of consecutive dry days (CDD), which is the maximum number of consecutive days in a year with precipitation less than 1 mm [8]. This indicator is widely used as an index for assessing seasonal drought and monitoring change in climatic extremes world-wide [9]. Drought is defined here as the “deficiency of precipitation relative to what is expected that, when extended over a season or a longer period of time, results in the inability to meet the demand of human activities and environment” [10]. Recurrent drought events, in addition to have direct consequences on hydration and health of the population, will also force households to search for continuous but potentially less safe water sources [11].

We calculated the average CDD over the years of the historical or future period, allowing spells of consecutive dry days to span over consecutive years. Results are shown in Figure S6, showing that that Sudan had to face droughts regularly and on average for 3 months to a year, between 1970 and 2006. The northern part of Sudan suffered droughts that lasted on average more than 5 years. According to future climate projections, droughts duration will increase and almost all of northern Sudan will endure droughts lasting more than one year on average.

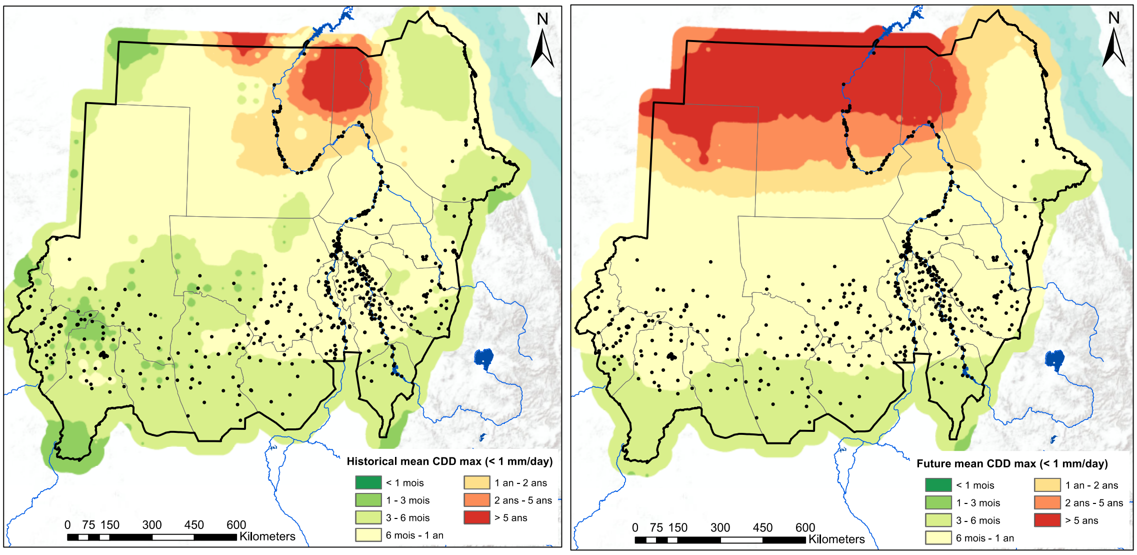

Figure S6 – Interpolation of historical (left) and future (right) average consecutive dry days per year

DWSI variable "Inter-annual variability"

This variable is based on the coefficient of variation (CV) of interannual precipitation, which is an indicator of precipitation variability over the years and is the standard deviation divided by the average annual rainfall. Irregular rainfall from year to year makes water management very difficult and requires water storage capacities which increases risk of water contamination [3]. $CV_{interannual}$ is calculated as follows:

$$CV_{interannual} = \frac{\sigma}{\bar{x}} \times 100$$

Where σ is the standard deviation (mm) and $\bar{x}$ the long-term mean of the amount of annual precipitation (mm).

The higher the CV (expressed in %), the more variable the year-to-year rainfall is. Stations for which the mean annual precipitation is 0 have a CV of 0. Results are shown in Figure S7, where we see that Northern, River Nile, El Gazira and Red Sea states were the most exposed to high variability in precipitation over the years during the historical period with a CV of more than 100%. In the future,

while southern states appear to have more stable annual precipitation, northern states, will experience greater fluctuations in year to year precipitation amount.

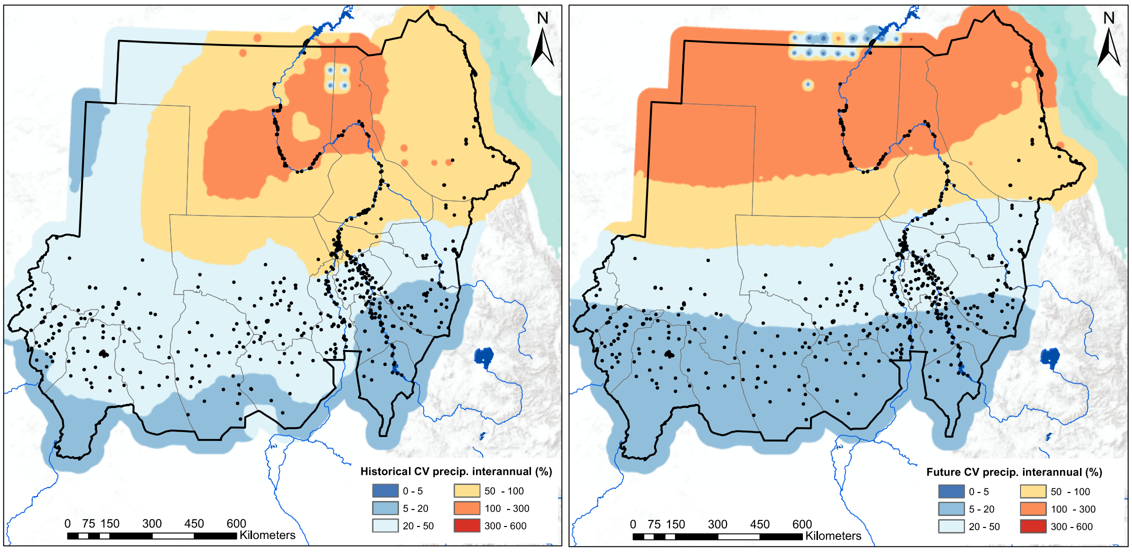

Figure S7 – Interpolation of historical (left) and future (right) coefficient of variation (CV) of annual precipitation

DWSI variable "Seasonal variability"

This variable is based on the coefficient of variation (CV) of intra-annual precipitation, which is an indicator of precipitation seasonality, namely the variability through one year and is calculated by the standard deviation divided by the average monthly rainfall. High seasonality implies that precipitation will be essentially short but intense, concentrated during two or three months. The increase of intra-annual precipitation variability may lead to change in groundwater recharge rate. In hydrophobic soils such as in the Sahel, infiltration during heavy rainfall is low [12]. $CV_{intraannual}$ is calculated as follows:

$$\boldsymbol{CV_{intraannual} = \frac{\sum_{i=1}^{n} \frac{\sigma_i}{\overline{x_i}}}{n} \times 100}$$

where σ_i is the standard deviation (mm) and $\overline{x_i}$ the mean of the amount of monthly precipitation (mm) for year $\boldsymbol{i}$. The $CV_{intraannual}$ is the mean of the n years and will be expressed in percentage. The higher the CV, the higher the precipitation seasonality.

As shown in Figure S8, Sudan has highly seasonal precipitation, with a rainy season limited to 2-3 months in the arid and semi-arid region and 3-4 months in the southern part, and rainfall occurs in isolated showers variable in duration and location [13]. The intra-annual CV is greater than 100%, which illustrates a high variability of precipitation throughout the years. In the future, this high variability is projected to increase in northern states, with CV of more than 200% over half of the country (Figure S8).

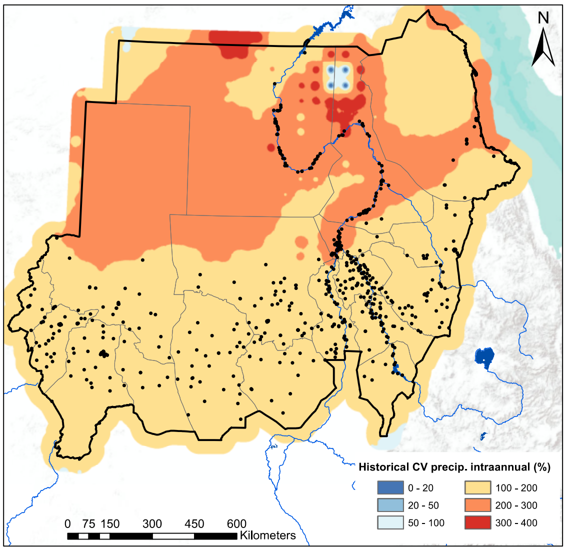

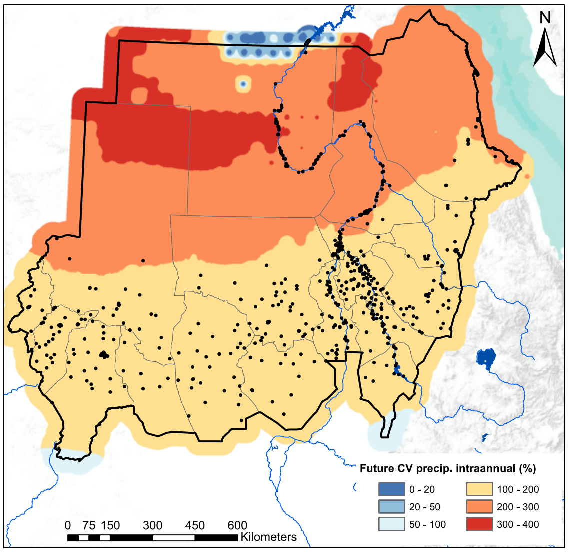

Figure S8 – Interpolation of historical and future coefficient of variation of monthly precipitation

DWSI variable "water balance ratio"

The water balance ratio is an indicator of groundwater recharge potential. Precipitation is the main source of groundwater recharge [14], but infiltration rate is highly dependent on evapotranspiration, which is a combination of surface water evaporation and vegetation transpiration. Therefore, we added in the DWSI calculation, the average ratio between precipitation and potential evapotranspiration (PET) in order to assess the amount of water available to recharge the aquifer. $WB\ ratio$ is computed as follows:

$$\boldsymbol{WB\ ratio} = \frac{\sum_{i=1}^{n} \frac{\boldsymbol{Pcp_{annual,i}}}{\boldsymbol{PET_{annual,i}}}}{\boldsymbol{n}}$$

Where Pcp_{annual} is the amount of annual precipitation (mm) and PET_{annual} in the amount of annual potential evapotranspiration (mm) for year i. The $WB\ ratio$ is the average of the n years.

To calculate the potential evapotranspiration, we uses the simple Thornthwaite equation, which required only the monthly-mean temperature and the latitude of the weather station [15]. This approach has been also applied in the widely used Standardized Precipitation Evapotranspiration Index (SPEI) [16], which is a drought index based exclusively on precipitation and temperature data.

$$\boldsymbol{PET_{annual}} = \sum_{j=1}^{12} \boldsymbol{PET_j} = \sum_{j=1}^{12} \boldsymbol{16K_j \left(\frac{10T_j}{I}\right)^m}$$

Where j is the months, T_j is the monthly mean temperature (°C), and I is the Annual Heat Index calculated as the sum of Monthly Heat Indices i_j:

$$\boldsymbol{I} = \sum_{j=1}^{12} \boldsymbol{i_j} = \sum_{j=1}^{12} \boldsymbol{\left(\frac{T_j}{5}\right)^{1.5}}$$

m is a coefficient depending on I calculated as:

$$\boldsymbol{m = (6.7 \times 10^{-7})I^3 - (7.7 \times 10^{-5})I^2 + (1.8 \times 10^{-2})I + 0.49}$$

and K_j is a correction coefficient which is a function of both latitude and month:

$$K_j = \left(\frac{N_j}{12}\right) \times \left(\frac{d_j}{30}\right)$$

Where d_j is the number of days and N_j is the theoretical number of daylight hours for month j, calculated as:

$$N_j = \left(\frac{24}{\pi}\right) arccos\left\{-tan\varphi \times tan\left[0.4093sen\left(\frac{2\pi J}{365} - 1.405\right)\right]\right\}$$

Where φ is the latitude in radians and J is the average Julian day of month j.

As shown in Figure S9, the average historical annual ratio between precipitation and potential evapotranspiration ranges from 0 to 1 over the whole of Sudan, meaning that evapotranspiration is always greater than precipitation. With future projected climate and the increase of average temperature, the ratio is going to be lower, meaning that less precipitation will be available for groundwater recharge or drinking water storage.

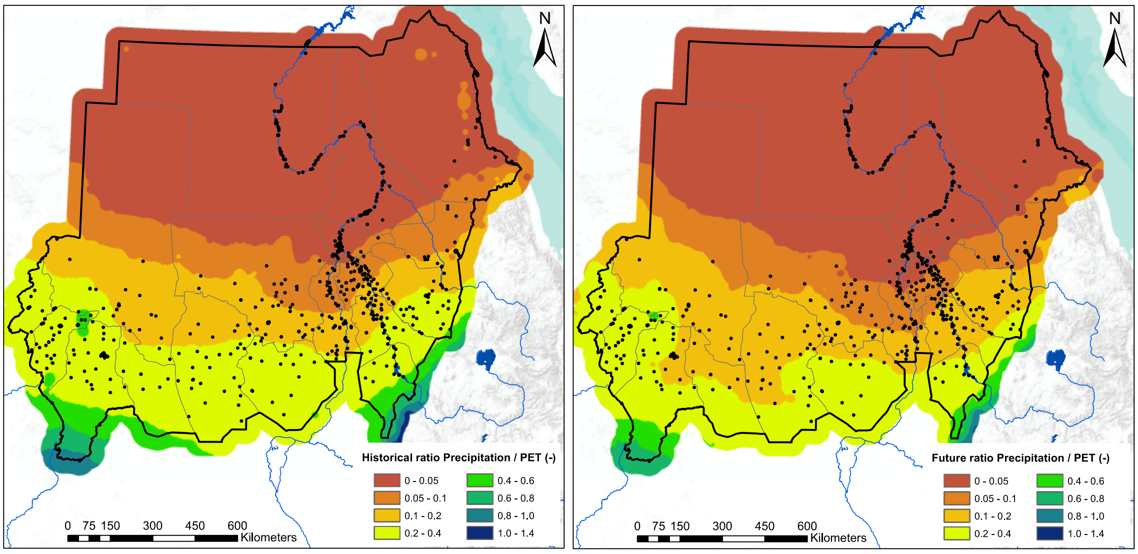

Figure S9 – Interpolation of historical and future water balance ratio calculated with annual precipitation and evapotranspiration

Spatial interpolation

As we have daily climate data and calculated variables at 0.5° grid points, we applied spatial interpolation to cover all Sudan with continuous data. DeGaetano et al. [17] concluded that there are no major differences to make such interpolation before or after climatic variables calculation. Therefore, we first calculated the four climatic variables at grid points before the spatial interpolation in order to reduce computing time and errors and to avoid the creation of large dataset.

To interpolate the climate variables, we used the Inverse Distance Weighted (IDW) method. This was preferred over other deterministic or geostatistical interpolation techniques (e.g., Spline or Kriging methods) for several reasons. First, for dense and regular network of sampled points, IDW can perform as well as complicated interpolation technics, even with high rainfall events [18]. Second, Sudan has moderate topographic variability, with a vast desert plateau, and thus there is less impact of altitude variation in the climate parameters interpolation [19]. Third, several studies have compared different interpolation methods for climate data in regions similar to Sudan and have found that IDW can perform even better than more complex geostatistical interpolation technics [17,18,20-23]. Finally, IDW is a fast and widely used method, readily available in most GIS software and does not required complicated parameterization [24]. This makes it easier to be applied in other contexts without the need of extensive and time-consuming preprocessing.

The IDW method is a deterministic and exact interpolation technique which determines the value of an attribute at unsampled points by making a weighted average of the values at sampled points, where the weighting is an inverse function of the Cartesian distance from point of interest to the sampled

points. Thus, sampled points closer to the unsampled location are more important in determining its value [25]. It is computed as follows:

$$Z_j = \left(\sum_i \frac{Z_i}{d_{ij}^n}\right) \Big/ \left(\sum_i \frac{1}{d_{ij}^n}\right)$$

Where Z_j is the estimated value for an unsampled point j, Z_i is the value of the sampled point i, namely here a climate station, d_{ij}^n is the distance between points i and j, with n the power parameter that determines the significance of the distance and set in this study at 2.

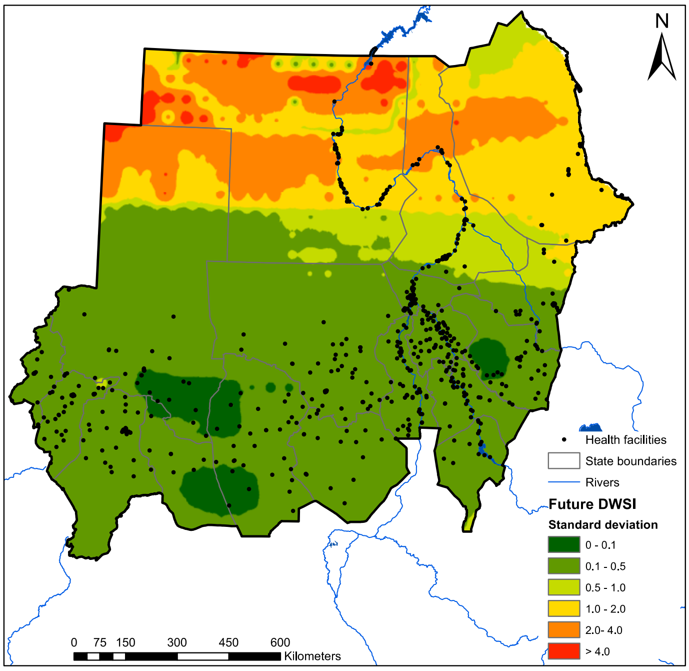

Figure S10 – Standard deviation in the future DWSI over the values obtained by running the five different GCMs

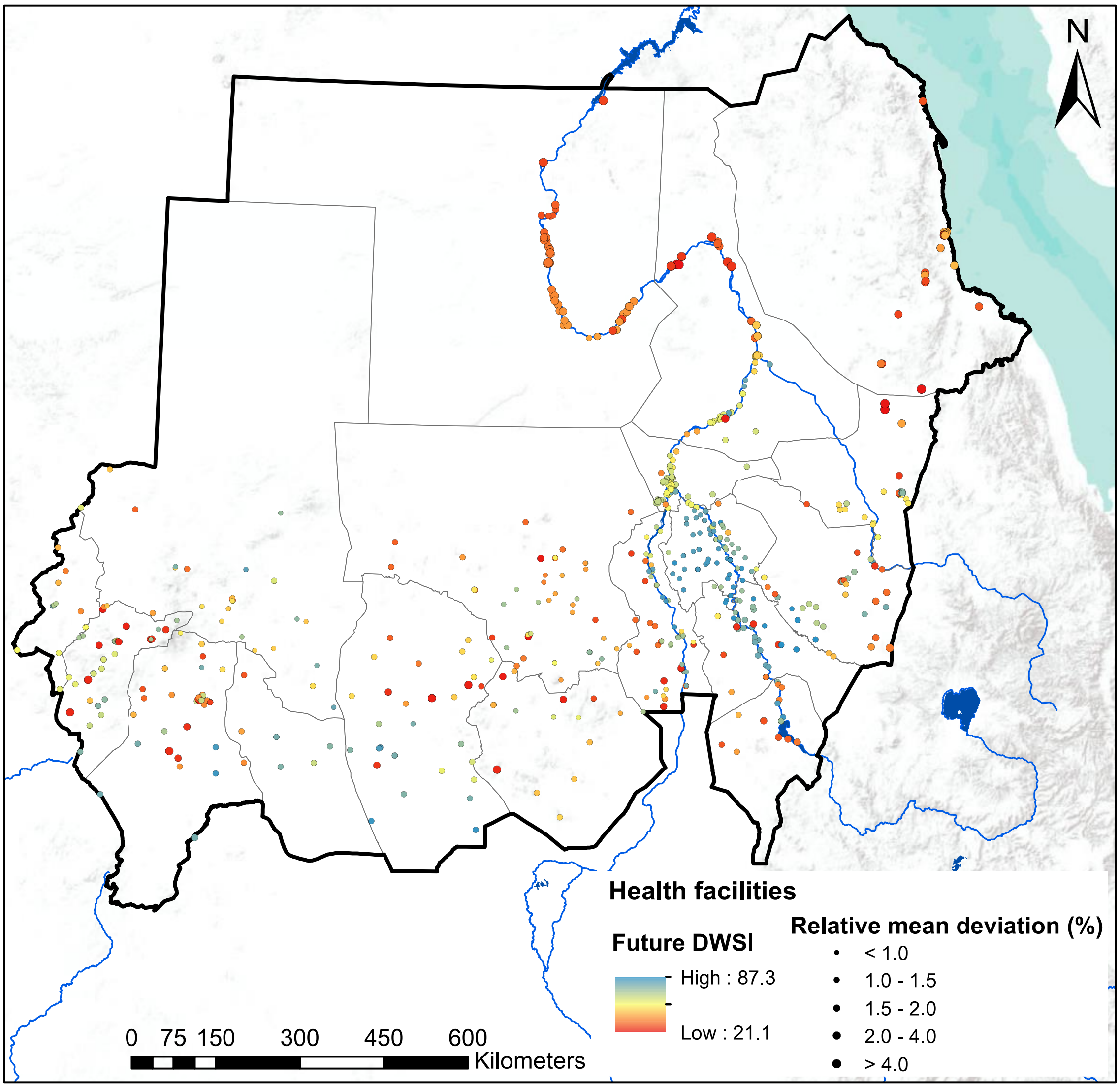

Figure S11 - Future DWSI at health facility level with the uncertainty range of the future DWSI (calculated over the 5 climatic models)

Table S1. Source, type, format, resolution, and year of each input dataset

Data layer	Source	Data format	Resolution	Year	Access data
Digital Elevation Model (DEM)	ASTER-GDEM	Raster	100 m	2015	Link https://asterweb.jpl.nasa.gov/gdem.asp 27 October 2022
Population distribution (density)	WorldPop	Raster	100 m	2010	Link https://hub.worldpop.org/geodata/listing?id=69 27 October 2022
Land cover/use with associated legend table	Copernicus	Raster	100 m	2015	Link https://land.copernicus.eu/global/products/lc 27 October 2022
Health facilities location and capacity	Sudan EmONC Need Assessment	Vector	-	2017	No open access
Barriers of movements (waterways, prohibited area)	Sudan national dataset	Vector	-	2018	No open access
Administrative boundaries	Sudan national dataset	Vector	-	2018	No open access
Road network with different classes	Sudan national dataset and OCHA	Vector	-	2018	No open access
Traveling speed scenarios	Established during UNFPA workshops	Table	-	2019	?

Table S2. Population and water supply statistics at State level

Source: Sudan Central Bureau of Statistics and World Bank - MICS 2010, MICS 2014, Statistics year book of the year 2009, Statistical year book of the year 2016, WB dataset

				Percentage of Sudan/State/region population (%)									
	Percentage of Sudan population (%) by state and region		Population growth rate	By mode of living in 2009			using improved water source		Using unimproved water source but appropriate water treatment		At less than 30 min fetching time (improved/unimproved)	
	2009	2014	2008 - 2018	Nomadic	Rural	Urban	2010	2014	2010	2014	2010	2014
**SUDAN**	31 797 000	37 289 000	2.41	9.1	58	32.9	60.5	68.0	0.9	4.1	77.6	62.4
**State**												
Northern	2.3	2.2	2.80	2.5	81.1	16.5	91.0	93.9	1.8	7.7	91.0	95.0
River Nile	3.6	3.6	2.10	2.6	68.5	28.9	78.0	88.3	0.1	1.2	83.3	91.4
Red Sea	4.5	3.8	2.25	18.5	42.7	38.9	27.4	33.2	0.8	8.5	78.9	28.4
Kassala	4.4	5.9	1.53	11.1	62.8	26.1	48.0	57.2	0.4	3.2	80.3	56.9
Gadarif	5.8	4.9	2.61	1.4	70.3	28.3	27.9	27.6	4.6	8.0	84.1	72.7
Khartoum	17.1	18.3	2.73	0.0	19.0	81.0	72.7	86.9	0.4	1.1	98.0	87.5
Gezira	11.6	11.9	2.48	0.1	81.0	18.9	79.2	88.9	0.5	11.5	93.0	92.9
White Nile	5.6	5.8	2.36	2.3	64.4	33.3	38.5	32.8	1.1	4.9	91.8	54.8
Sinnar	4.2	4.4	2.43	2.1	76.4	21.5	60.7	88.8	1.5	2.8	89.5	60.9
Blue Nile	2.7	2.7	2.81	3.9	71.9	24.2	39.9	71.3	0.4	1.0	66.8	59.1
North Kordofan	6.6	6.3	2.55	13.1	67.2	19.7	53.8	69.9	0.1	1.7	71.7	55.9
South Kordofan	2.8	2.7	2.68	12.0	64.7	23.4	49.7	60.0	0.8	0.1	62.7	49.4
West Kordofan	4.6	4.4	2.48	12.0	64.7	23.4	49.7	86.0	0.8	0.0	62.7	38.9
North Darfur	6.8	6.0	2.40	19.2	64.0	16.8	59.8	50.7	0.6	4.3	54.7	21.0
West Darfur	2.4	2.4	2.50	18.4	64.6	17.0	44.5	67.6	0.3	1.6	57.3	44.8
South Darfur	9.3	9.5	2.05	24.2	54.5	21.3	69.4	46.6	0.0	1.9	45.7	32.7
Central Darfur	1.8	1.8	2.62	18.4	64.6	17.0	44.5	50.8	0.3	0.7	57.3	42.2
East Darfur	3.9	3.5	2.12	24.2	54.5	21.3	69.4	45.1	0.0	5.2	45.7	43.2
**Area**												
Urban	42.0	29.8	2.78	-	-	-	66.6	78.3	0.7	3.7	95.8	75.4
Rural	58.0	70.2	2.05	-	-	-	57.7	63.5	0.9	4.2	68.9	56.5
**Education of the houshold head**												
None							55.8	59.7	8.0	4.0	71.6	51.6
Primary							62.9	71.0	0.6	5.1	82.4	65.4
Secondary							72.7	78.8	1.8	3.0	89.1	77.4
Higher							-	86.6	-	5.1	-	86.0
Missing							57.9	57.4	0.8	0.0	76.3	52.9
**Wealth index quintile**												
Poorest							61.5	45.4	0.0	2.9	43.7	25.1
Second							43.5	53.4	0.7	4.4	65.1	44.7
Middle							41.9	60.0	1.3	4.3	83.9	58.0
Fourth							63.0	85.2	1.2	6.4	95.2	87.1
Richest							92.8	96.1	1.2	7.0	99.2	97.0

References

1. FMoH; Central Bureau of Statistics; UNICEF. Multiple Indicator Cluster Survey 2014 of Sudan, Final Report. *UNICEF and Central Bureau of Statistics* **2016**.
2. FMoH; Central Bureau of Statistics; UNICEF. Sudan Household Health Survey - 2010. **2012**.
3. WHO. Guidelines for drinking-water quality: fourth edition incorporating the first addendum. **2017**.
4. Trevett, A.F.; Carter, R.C.; Tyrrel, S.F. Water quality deterioration: a study of household drinking water quality in rural Honduras. *International journal of environmental health research* **2004**, *14*, 273-283.
5. Howard, G.; Bartram (WHO), J. Domestic water quantity, service level and health. **2003**.
6. Central Bureau of Statistics. Statistical year book for the year 2016. **2017**.
7. MacDonald, A.M.; Bonsor, H.C.; Dochartaigh, B.É.Ó.; Taylor, R.G. Quantitative maps of groundwater resources in Africa. *Environmental Research Letters* **2012**, *7*, 024009.
8. Frich, P.; Alexander, L.V.; Della-Marta, P.; Gleason, B.; Haylock, M.; Tank, A.K.; Peterson, T. Observed coherent changes in climatic extremes during the second half of the twentieth century. *Climate research* **2002**, *19*, 193-212.
9. Duan, Y.; Ma, Z.; Yang, Q. Characteristics of consecutive dry days variations in China. *Theoretical and Applied Climatology* **2017**, *130*, 701-709.
10. Hayes, M.; Svoboda, M.; Wall, N.; Widhalm, M. The Lincoln declaration on drought indices: universal meteorological drought index recommended. *Bulletin of the American Meteorological Society* **2011**, *92*, 485-488.
11. Pattanayak, S.K.; Yang, J.C.; Whittington, D.; Bal Kumar, K. Coping with unreliable public water supplies: Averting expenditures by households in Kathmandu, Nepal. *Water Resources Research* **2005**, *41*.
12. Kundzewicz, Z.W.; Doell, P. Will groundwater ease freshwater stress under climate change? *Hydrological Sciences Journal* **2009**, *54*, 665-675.
13. FAO. AQUASTAT database. Available online: http://www.fao.org/nr/water/aquastat/countries_regions/SDN/indexfra.stm (accessed on 21.02.19).
14. Lanzoni, M.; Darling, W.; Edmunds, W. Groundwater in Sudan: An improved understanding of wadi-directed recharge. *Applied geochemistry* **2018**, *99*, 55-64.
15. Thornthwaite, C.W. An approach toward a rational classification of climate. *Geographical review* **1948**, *38*, 55-94.
16. Vicente-Serrano, S.M.; Beguería, S.; López-Moreno, J.I. A multiscalar drought index sensitive to global warming: the standardized precipitation evapotranspiration index. *Journal of climate* **2010**, *23*, 1696-1718.
17. DeGaetano, A.T.; Belcher, B.N.; Noon, W. Temporal and spatial interpolation of the standardized precipitation index for computational efficiency in the dynamic drought index tool. *Journal of Applied Meteorology and Climatology* **2015**, *54*, 795-810.
18. Camera, C.; Bruggeman, A.; Hadjinicolaou, P.; Pashiardis, S.; Lange, M.A. Evaluation of interpolation techniques for the creation of gridded daily precipitation (1× 1 km2); Cyprus, 1980–2010. *Journal of Geophysical Research: Atmospheres* **2014**, *119*, 693-712.
19. Rhee, J.; Carbone, G.J.; Hussey, J. Drought index mapping at different spatial units. *Journal of Hydrometeorology* **2008**, *9*, 1523-1534.
20. Keblouti, M.; Ouerdachi, L.; Boutaghane, H. Spatial interpolation of annual precipitation in Annaba-Algeria-comparison and evaluation of methods. *Energy Procedia* **2012**, *18*, 468-475.
21. Dobesch, H.; Dumolard, P.; Dyras, I. *Spatial interpolation for climate data: the use of GIS in climatology and meteorology*; John Wiley & Sons: 2013.
22. Rhee, J.; Carbone, G.J. Estimating drought conditions for regions with limited precipitation data. *Journal of Applied Meteorology and Climatology* **2011**, *50*, 548-559.
23. Akhtari, R.; Morid, S.; Mahdian, M.H.; Smakhtin, V. Assessment of areal interpolation methods for spatial analysis of SPI and EDI drought indices. *International Journal of Climatology: A Journal of the Royal Meteorological Society* **2009**, *29*, 135-145.
24. Dyras, I.; Dobesch, H.; Grueter, E.; Perdigao, A.; Tveito, O.E.; Thornes, J.E.; van der Wel, F.; Bottai, L. The use of Geographic Information Systems in climatology and meteorology: COST 719. *Meteorological Applications* **2005**, *12*, 1-5.

25. Shepard, D. A two-dimensional interpolation function for irregularly-spaced data. In Proceedings of the Proceedings of the 1968 23rd ACM national conference, 1968; pp. 517-524.
